# LH and hCG Action on the Same Receptor Results in Quantitatively and Qualitatively Different Intracellular Signalling

**DOI:** 10.1371/journal.pone.0046682

**Published:** 2012-10-05

**Authors:** Livio Casarini, Monica Lispi, Salvatore Longobardi, Fabiola Milosa, Antonio La Marca, Daniela Tagliasacchi, Elisa Pignatti, Manuela Simoni

**Affiliations:** 1 Department of Biomedical, Metabolic and Neural Sciences, University of Modena and Reggio Emilia, Modena, Italy; 2 Center for Genomic Research, University of Modena and Reggio Emilia, Modena, Italy; 3 Medical Liaison Office, Merck Serono S.p.A., Rome, Italy; 4 Mother-Infant Department, Institute of Obstetrics and Gynecology, University Hospital of Modena, Modena, Italy; 5 Azienda USL di Modena, Modena, Italy; University of Muenster, Germany

## Abstract

Human luteinizing hormone (hLH) and chorionic gonadotropin (hCG) act on the same receptor (LHCGR) but it is not known whether they elicit the same cellular and molecular response. This study compares for the first time the activation of cell-signalling pathways and gene expression in response to hLH and hCG. Using recombinant hLH and recombinant hCG we evaluated the kinetics of cAMP production in COS-7 and hGL5 cells permanently expressing LHCGR (COS-7/LHCGR, hGL5/LHCGR), as well as cAMP, ERK1/2, AKT activation and progesterone production in primary human granulosa cells (hGLC). The expression of selected target genes was measured in the presence or absence of ERK- or AKT-pathways inhibitors. In COS-7/LHCGR cells, hCG is 5-fold more potent than hLH (cAMP ED_50_: 107.1±14.3 pM and 530.0±51.2 pM, respectively). hLH maximal effect was significantly faster (10 minutes by hLH; 1 hour by hCG). In hGLC continuous exposure to equipotent doses of gonadotropins up to 36 hours revealed that intracellular cAMP production is oscillating and significantly higher by hCG versus hLH. Conversely, phospho-ERK1/2 and -AKT activation was more potent and sustained by hLH versus hCG. ERK1/2 and AKT inhibition removed the inhibitory effect on *NRG1* (neuregulin) expression by hLH but not by hCG; ERK1/2 inhibition significantly increased hLH- but not hCG-stimulated *CYP19A1* (aromatase) expression. We conclude that: i) hCG is more potent on cAMP production, while hLH is more potent on ERK and AKT activation; ii) hGLC respond to equipotent, constant hLH or hCG stimulation with a fluctuating cAMP production and progressive progesterone secretion; and iii) the expression of hLH and hCG target genes partly involves the activation of different pathways depending on the ligand. Therefore, the LHCGR is able to differentiate the activity of hLH and hCG.

## Introduction

Luteinizing hormone (LH) and chorionic gonadotropin (CG) are heterodimeric glycoprotein hormones acting on the same receptor, the luteinizing hormone-chorionic gonadotropin receptor (LHCGR) [1], which is found as dimer/oligomer at the cell membrane [2]. LH is the physiological hormone in non-pregnant women, produced by the pituitary in a pulsatile fashion. LH binds to LHCGR on the granulosa cells surface, resulting in progesterone production, ovulation, luteinization and corpus luteum formation [3]. Moreover, LH stimulates androstenedione and testosterone production in theca cells. In the human ovary, androstenedione is aromatized to estrone by granulosa cells and finally converted to estradiol by 17-β-hydroxysteroid dehydrogenase type I, representing the system known as the two-cell-two-gonadotropin regulation of estrogen synthesis [3]. After the ovulation, LH supports the transient life span of the corpus luteum acting on the luteinized granulosa cells [3]. Conversely, CG is a hormone produced mainly by placental trophoblast cells during pregnancy in an increasing, non-pulsatile fashion [4]. Apart from equine CG (eCG), which mediates a predominant FSH-like activity [5], CG with exclusive, unique LH-like activity exists only in primates and its β-subunit gene is present in increasing copy number, according to the increasing structural complexity of placental implantation in the primate species [6].

Human LH (hLH) and CG (hCG) differ in their half-life (60–120 minutes for hLH, several hours for hCG [7]–[13] and in some structural features, such as the presence of a carboxyl terminal peptide (CTP) and the type and amount of glycosylation. Due to this heterogeneity and derivation from extractive preparations, gonadotropins have been difficult to quantify accurately in the past, and most *in vitro* experiments have been conducted using urinary hCG calibrated by *in vivo* bioassay against standard preparations expressed in activity units [14]. With the advent of recombinant gonadotropins, highly homogeneous and consistent r-hLH and r-hCG can be accurately quantified in molar terms [15] and used to compare their effects *in vitro* at exactly equimolar concentrations.

Being structurally different, it should be expected that hLH and hCG display different hormone-receptor interaction features and, consequently, might be not equivalent at molecular and cellular level. There are some hints that hLH and hCG may not have the same activity. Some residues of the LHCGR extracellular domain are indeed able to differentiate binding of hLH and hCG [16] and the human LHCGR can react differently to hLH and hCG when exon 10 is lacking (LHCGR-10) [17]. LHCGR-10 can bind either gonadotropin with similar affinity but cAMP production is drastically impaired upon hLH but not hCG stimulation [17], [18], suggesting that exon 10 of the LHCGR can distinguish between hLH and hCG. Whether this translates physiologically into preferential activation of different signal transduction pathways and, eventually, different cell responses, is not known.

Some LHCGR-dependent effects are mediated by the activation of the cyclic AMP-protein kinase A (cAMP/PKA) pathway, which stimulates progesterone production and has been associated to morphological changes [19] and apoptosis [20], [21] in granulosa cells. However, additional signalling pathways (e.g. AKT- and ERK1/2-pathways) are involved [1] in LHCGR-dependent events such as proliferation, differentiation and survival [22], for example *via* expression of EGF-like growth factors [23]. Lastly, aromatase expression and steroidogenic function via LHCGR activation are likely to involve cAMP/PKA, ERK1/2 and AKT pathways, all playing a crucial role in the final stages of maturation of human oocytes and follicles [24], [25].

While equivalence of hLH and hCG in activating the downstream signaling of LHCGR is presumed, this issue has never been investigated thoroughly so far. In this work, after confirming that extractive and recombinant gonadotropins are equivalent in essence as far as their effects in vitro are concerned, we evaluated the effects of hLH and hCG on the cell response by systematically assessing the activation of cAMP/PKA-, ERK1/2- and AKT-pathways in three validated *in vitro* models consisting of a COS-7 cell line permanently transfected with human LHCGR (COS-7/LHCGR) [18], the immortalized human granulosa cell line hGL5 [26] permanently transfected with the human LHCGR (hGL5/LHCGR) and human primary granulosa cells (hGLC) [27]. The COS-7/LHCGR cell system is a standardized model, constitutively expressing a fix number of LHCGR under the transcriptional regulation of the cytomegalovirus promoter [18]. Conversely, primary hGLC obtained from women undergoing assisted reproduction procedures naturally express the LHCGR, which is presumably subjected to “physiological” control of LHCGR gene transcription and different modulation of the desensitization mechanisms [28]. Finally, the hGL5 cell line derives from human granulosa cells immortalized by transformation with the E6 and E7 regions of human papillomavirus. Despite their steroidogenic activity, hGL5 cells do not naturally express gonadotropin receptors [26] and were permanently transfected with the human LHCGR for this study. These cell model were used to assess the kinetics of cAMP and progesterone production and to investigate some intracellular events following acute and chronic exposure to equipotent hLH and hCG doses. Remarkable differences in LH and hCG action were demonstrated.

## Methods

A detailed description of the methods is available as Methods S1.

### Recombinant and extractive gonadotropins

Human, highly purified recombinant LH (r-hLH; Luveris) and CG (r-hCG; Ovitrelle) were kindly provided by Merck-Serono S.p.A. (Rome, Italy). LH extracted from human pituitary (ex-hLH) and CG extracted from human pregnancy urine (ex-hCG) were purchased by Sigma-Aldrich (Sigma-Aldrich, St. Louis, MO).

### Cell lines

A COS-7 cell line permanently transfected with LHCGR wild type (COS-7/LHCGR) was kindly provided by Prof. Gromoll (Centre for Reproductive Medicine and Andrology, University of Münster, Germany). The immortalized human granulosa cell line hGL5 [26] was permanently transfected by electroporation with LHCGR wild type. For stable transfection we used the pTracer vector (Invitrogen, Leek, The Netherlands), containing the cytomegalovirus promoter in front of the multiple cloning site and the green fluorescent protein reporter gene [18]. COS-7/LHCGR cells were cultured in DMEM supplemented with 10% FBS, 2 mM L-glutamine, 100 U/ml penicillin and 100 µg/ml streptomycin. This cell line, overexpressing the human LHCGR, was extensively validated previously [18]. hGL5/LHCGR cells were cultured in DMEM/F12 supplemented with 10% FBS, 2% Ultroser G, 2 mM L-glutamine, 100 U/ml penicillin and 100 µg/ml streptomycin. All cell lines were maintained in an incubator at 37°C and with 5% CO_2_.

### Granulosa-lutein cell isolation and culture

Human primary granulosa-lutein cells (hGLC) were isolated from ovarian follicles of women undergoing oocyte retrieval for assisted reproduction technologies (ART). The cells from 3–4 different patients were pooled and collected using a 50% Percoll gradient (GE Healthcare, Little Chalfont, UK) following a procedure previously described [27], then cultured in a CO_2_ cell incubator. Before each experiment, granulosa cells were maintained in culture until day 6, to allow the recovery of the response to gonadotropins (Fig. S1).

### Patients selection

Women undergoing ovarian stimulation for infertility due to tubal or male factor were included. Study approval was obtained from the local ethics committee and informed, written consent was obtained from each patient. (Ethics committee approval - dossier number: Pratica 161/11, date: 21 september 2011, Prot. n. 3186/C.E. Comitato Etico Provinciale di Modena). The patients had to match the following criteria: absence of endocrine abnormalities; absence of severe viral or bacterial infections; age between 25–45 years.

### cAMP stimulation protocols

For cAMP stimulation a validated protocol was followed [27]. The COS-7/LHCGR, hGL5/LHCGR and granulosa cells were seeded in triplicate in 24-well plates for cAMP dose-response experiments and serum starved 12 hours before the experiments. Cells were stimulated using increasing doses of r-hLH, r-hCG, ex-hLH or ex-hCG as appropriate (ranging between 0.1 pM-1 mM) in the presence of 500 µM IBMX (Sigma-Aldrich). Total cAMP was measured after 3 hours incubation and the cAMP ED_50_ values for hLH and hCG were calculated. A total of 4 independent experiments were performed.

To evaluate the kinetics of response to continuous exposure to gonadotropins, time-course experiments were performed. The COS-7/LHCGR and hGLC were stimulated using the cAMP equipotent ED_50_ doses of r-hLH or r-hCG, for different times ranging between 5 minutes and 36 hours in the presence of IBMX. The intracellular cAMP was measured after each incubation. Cell viability was also evaluated [29]. A total of 3 independent experiments were performed.

The quantitative detection of cAMP was performed in triplicate by a competitive ELISA kit and the data were entered into a curve fitting software which extrapolates the cAMP concentration against a standard curve. The data were represented using a log regression analysis.

### Immunofluorescence analysis of human granulosa cells

Immunofluorescence analysis was performed to evaluate the kinetics of receptor internalization resulting from continuous *in vitro* stimulation of hGLC by gonadotropins. Serum-starved hGLC were stimulated for different times with the ED_50_ dose of hLH or hCG. After stimulation, the cells were fixed and sequentially incubated with anti-LHCGR [30] and anti-ERK1/2 antibody to be observed in confocal microscopy. Negative and positive controls for the antibodies and non-permeabilized cells were also included.

### Phospho-ERK1/2 and phospho-AKT stimulation and Western blot analysis

To evaluate the doses of r-hLH, r-hCG, ex-hLH or ex-hCG resulting in the maximum level of stimulation of ERK1/2- and AKT-pathway (ED_MAX_), dose-response experiments were performed. 12 hours serum-starved hGLC were stimulated for 15 minutes [31], [32] with increasing doses of r-hLH or r-hCG (ranging between 0.1 pM-1 mM), including negative controls. To compare the response to recombinant *versus* extractive gonadotropins also hGL5/LHCGR cells were used. In time-course experiments the cells were then stimulated over 1 hour with 100 pM r-hLH or r-hCG (ED_MAX_), previously obtained in dose-response experiments. Negative controls were included in each step of the time-course experiment. The cells were immediately lysed for protein extraction in 4°C-cold RIPA buffer added with protease and phosphatase inhibitors. A total of 4 independent experiments were performed by using each time a different pool of granulosa cells obtained from 3–4 different patients.

Phospho-ERK1/2 and phospho-AKT activation were evaluated by Western blot analysis after 12% SDS-PAGE. Equal protein loading was confirmed against total ERK1/2 after stripping the membranes. The signals were revealed by chemiluminescence, then acquired and semi-quantitatively evaluated by an image analysis system.

### Stimulation for gene expression analysis

Serum-starved hGLC were stimulated for 12 hours with r-hLH or r-hCG ED_MAX_. One-hour pre-incubated samples with ERK1/2- or AKT-pathway inhibitors were also included. After stimulation, total RNA was extracted and an equal amount in cDNA synthesis reactions was used.

### Real-time PCR analysis

Real-time PCR was performed in triplicates with the primers shown in Table 1. The *ribosomal protein S7* (*RPS7*) gene was used as normalizer. The thermal cycling settings for all genes were the following: 45 cycles of 30 s of melting at 95°C followed by 10 s of annealing and extension at 60°C. Normalized gene expression was evaluated using the 2^−ΔCt^ method [33] and expressed as fold increase over its unstimulated sample (basal level). A total of four experiments were performed.

**Table 1:** Primer sequences used in real-time PCR experiments.

Gene	Oligonucleotide sequences	Product length (bp)	NCBI Ref. Sequence	Protein name
AREG	F:GACACCTACTCTGGGAAGCG	121	NM_001657.2	Amphiregulin
	R:AAGGCATTTCACTCACAGGG			
EREG	F:TACTGCAGGTGTGAAGTGGG	139	NM_001432.2	Epiregulin
	R:TGGAACCGACGACTGTGATA			
NRG1*	F:CCCCGATTGAAAGAGATGAA	116	NM_001159999.1	Neuregulin 1
	R:TTCCCATTCTTGAACCACTTG			
CYP19A1	F:CCCTTCTGCGTCGTGTCAT	86	NM_000103.3	Aromatase
	R:GATTTTAACCACGATAGCACTTTCG			
RPS7**	F: AATCTTTGTTCCCGTTCCTCA	135	NM_001011.3	Ribosomal protein S7
	R: CGAGTTGGCTTAGGCAGAA			

* Primer chosen to include all NRG1 transcript variants, verified by NCBI BLAST.

** Endogenous control gene.

### Statistical analysis

Each value obtained from hLH and hCG-stimulated cells was normalized for the corresponding control value. In time-course experiments for ERK1/2 and AKT, the semi-quantitative evaluations were graphically expressed in relative units. Data are expressed as means ± SEM. Mann Whitney's *U*-test, unpaired T-test or two-way analysis of variance were performed where appropriate. In time-course experiments, each data-set was verified with D'Agostino and Pearson normality test (alpha = 0.05). Values were considered statistically significant for P<0.05.

## Results

### Short-term kinetics of cAMP production

**Figure 1:**
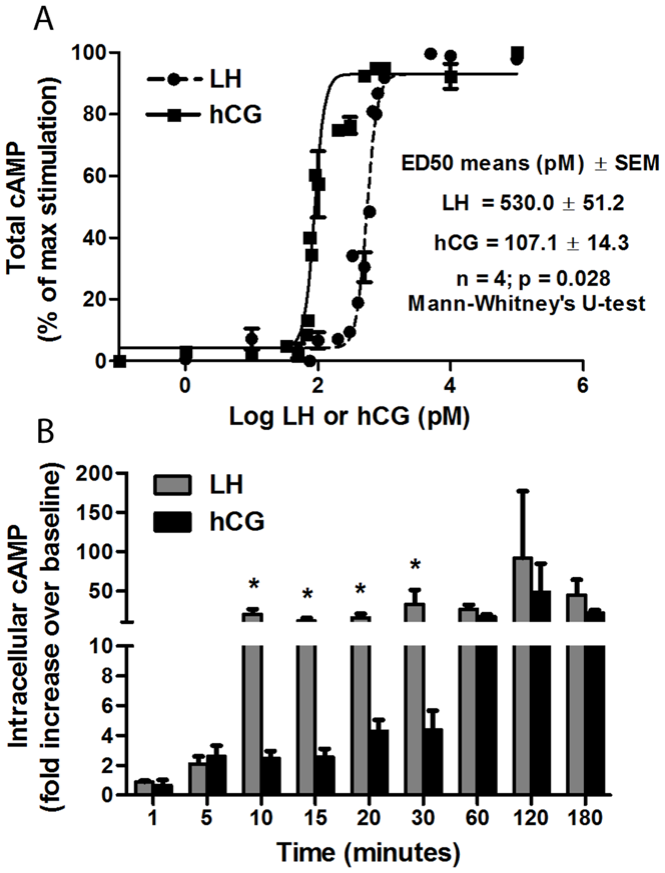
Dose-response and time-course experiments. **a.** Dose-response experiment to r-hLH and r-hCG in COS7/LHCGR in the presence of 500 µM IBMX. Total cAMP was measured after 3 hours of incubation and the results of four independent experiments were plotted (Mean±SEM). **b.** Time-course experiment performed by continuous incubation of COS7/LHCGR for different time-points in the presence of 500 µM IBMX and gonadotropins at ED_50_ doses (500 pM r-hLH; 100 pM r-hCG). Intracellular cAMP was measured (n = 3; Mean±SEM). Asterisk indicates the significant differences of hLH *vs* hCG (*t-test*; *p*<0.05).

To evaluate whether r-hLH and r-hCG stimulation of the human LHCGR results in the same kinetics of cAMP production we performed dose-response and time course-experiments using the COS-7/LHCGR cell line. This model allows testing the early effects of the two gonadotropins in “steady” receptor conditions, since no regulation of LHCGR gene transcription occurs in these stably transfected cells constitutively expressing the LHCGR under the control of the cytomegalovirus promoter. Dose-response studies were performed by measuring total cAMP after 3 hours of stimulation with increasing doses of hCG or hLH in the presence of IBMX (500 µM). A significant difference in the kinetics of total cAMP production was found, with dose-response curves shifted significantly to the left in the case of hCG. As shown in Fig. 1, the ED_50_ of r-hCG (107.1±14. 3 pM) resulted approximately 5 times lower than the ED_50_ of r-hLH (530.0±51.2 pM) (n = 4, p<0.05, Fig. 1A). Since recombinant gonadotropins could differ from pituitary hLH and urinary hCG in the extent and type of glycosylation this could affect cAMP production, making the results obtained with “artificial” gonadotropins potentially different from those with “natural” gonadotropins. To assess this, a comparison between both type of gonadotropins was performed in different cell models (Fig. S2). The results obtained were similar, indicating that recombinant and extractive gonadotropins result in comparable effects on activation of signaling pathways, as otherwise well known from clinical practice.

The ED_50_ doses of r-hLH and r-hCG were then used in time-course experiments, based on the measurement of intracellular cAMP in COS-7/LHCGR cells stimulated for up to 180 minutes by r-hLH (500 pM) or r-hCG (100 pM), in the presence of 500 µM IBMX (500 µM). Using equipotent doses, accumulation of intracellular cAMP by r-hLH resulted significantly faster, with maximal activation achieved in 10 minutes, while, by r-hCG, the same levels of maximal stimulation were attained only after 60 minutes of stimulation (Fig. 1B). Maximal intracellular cAMP accumulation was approximately 20–30 times higher than basal levels in untreated cells and was equally reached in both r-hLH- and r-hCG-stimulated cells, confirming that the ED_50_ doses were indeed equipotent in acute activation. After the first 60 min of continuous exposure to r-hLH or r-hCG intracellular cAMP concentrations remained elevated (Fig. 1B). Therefore, equimolar concentrations r-hLH and r-hCG possess significantly different *in vitro* potency (in terms of cAMP) and equipotent concentrations of r-hLH and r-hCG stimulate intracellular cAMP accumulation with significantly different kinetics.

When repeated in hGLC cells the cAMP dose-response and time course experiments confirmed the same difference between r-hLH and r-hCG (Fig. S3).

### Long-term kinetics of cAMP and progesterone production in hGLC continuously exposed to hLH or hCG

hGLC obtained at oocyte pickup in ART programs are a well-known, widely used model for the study of cellular response to gonadotropins [27] and constitute a more physiological model than COS-7/LHCGR cells, since they naturally express the LHCGR and display physiological control of the receptor gene transcription and downregulation *in vitro* [31]. In preliminary experiments we determined that hGLC do not respond significantly, in terms of cAMP production, to r-hLH or r-hCG for up to four days in culture but respond optimally on day 6 (Fig. S1), as previously described for FSH as well [27]. To compare the kinetics of intracellular cAMP beyond the first 60 min hGLC were stimulated on culture day 6 with ED_50_ doses of r-hLH or r-hCG for up to six hours. Intracellular cAMP levels decreased after the first three hours but were not extinguished by 360 min (Fig. S4).

**Figure 2:**
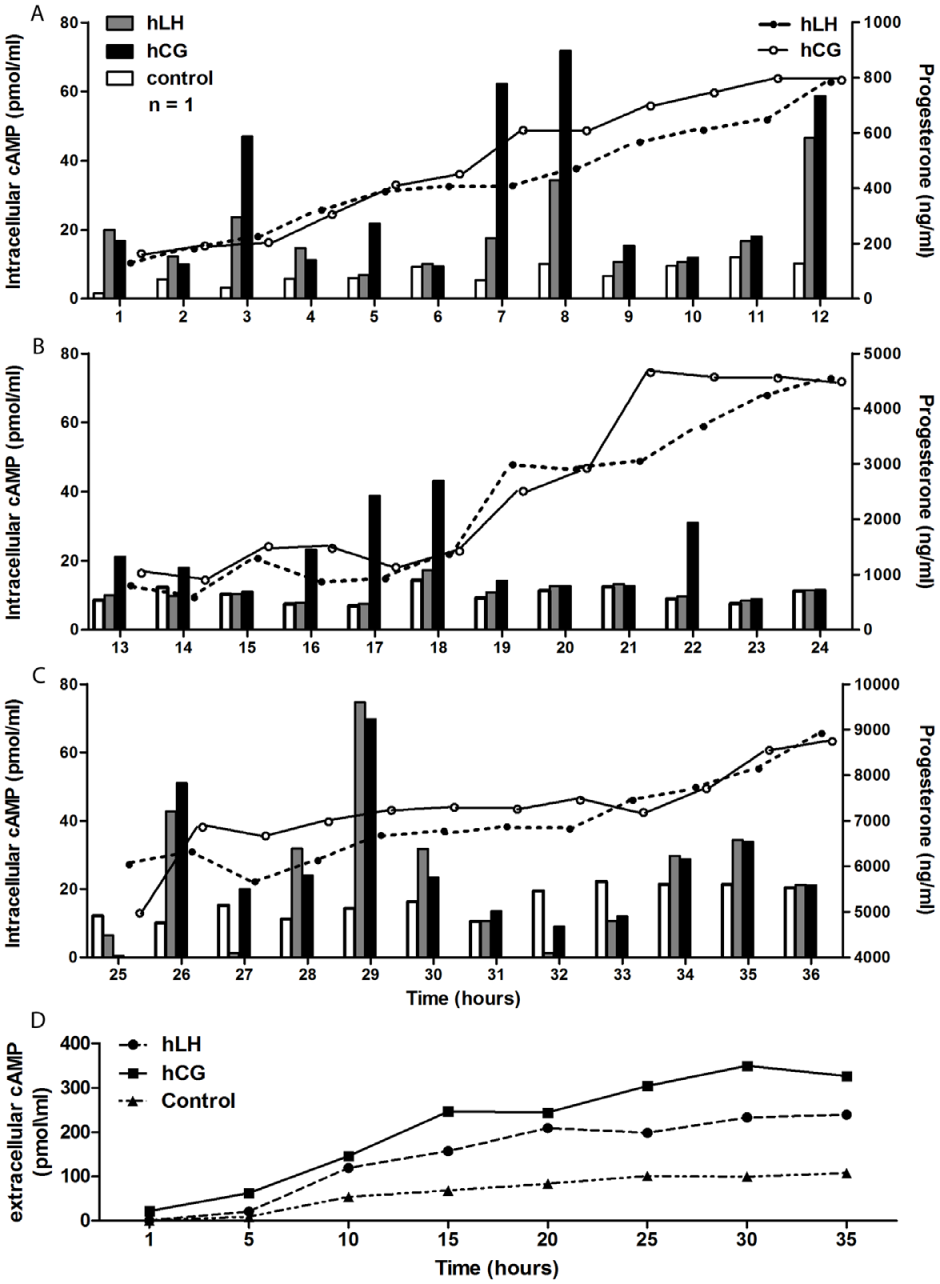
Long-term kinetics of intracellular cAMP production and extracellular progesterone and cAMP release in a primary hGLC sample continuously exposed to r-hLH or r-hCG. Absolute values of intracellular cAMP (bars) and extracellular progesterone (lines) production by hGLC cultured in the presence IBMX (500 µM) under continuous stimulation by hLH (500 pM) or hCG (100 pM) for up to 36 hours: (**a**) 0–12 hours, (**b**) 13–24 hours, (**c**) 25–36 hours. cAMP basal level of the unstimulated control cells is also shown for each time-point. **d.** Measurement of extracellular cAMP, released in culture medium. One representative of three independent measurement is shown for each graph. Please notice the different progesterone scale on the Y axis on right side.

**Figure 3:**
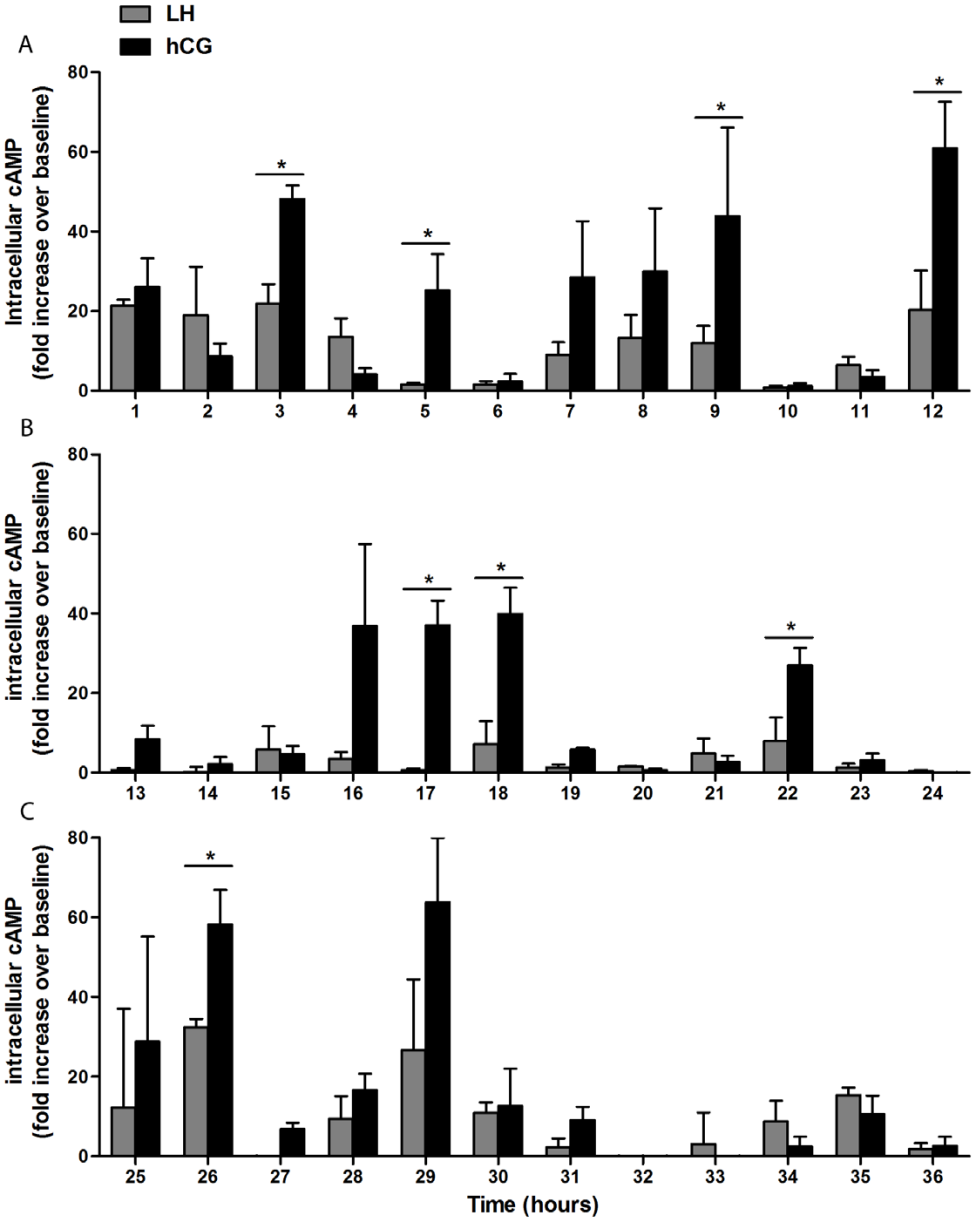
Long-term kinetics of intracellular cAMP production in hGLC continuously exposed to r-hLH or r-hCG. hGLC were cultured in the presence IBMX (500 µM) under continuous stimulation by r-hLH (500 pM) or r-hCG (100 pM) for up to 36 hours: (**a**) 0–12 hours, (**b**) 13–24 hours, (**c**) 25–36 hours. Results are means±SEM (n = 3). Asterisks indicate significant differences between hCG and hLH at the given time point (n = 3; *t-test*; *p*<0.05).

**Figure 4:**
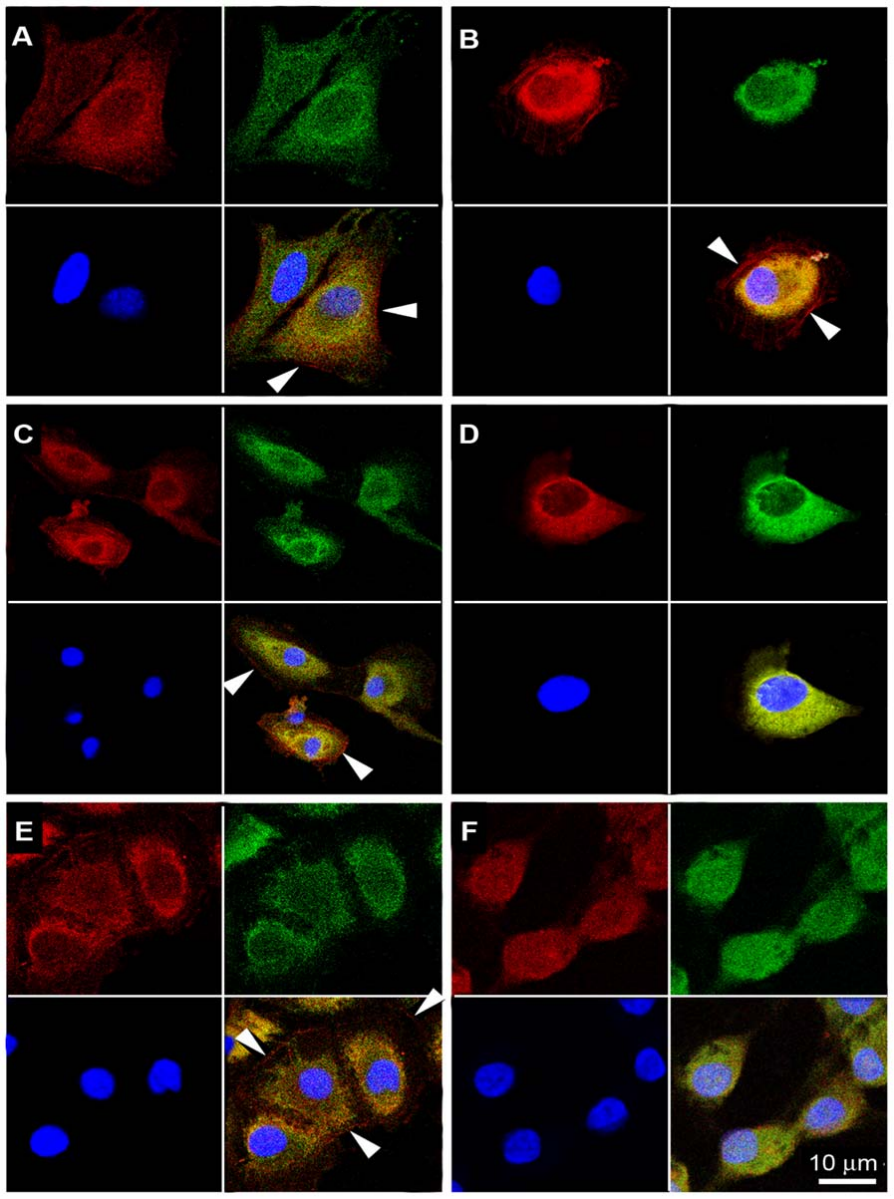
Cellular localization of LHCGR and morphological changes of hGLC under continuous exposure to hLH 500 (pM) by confocal microscopy. **a.** Unstimulated cells (control) after 1 hour. **b.** hLH-treated cells after 1 hour. **c.** Unstimulated cells after 15 hours. **d.** LHCGR sequestration from cell surface in hLH-treated hGLC, after 15 hours. **e.** Unstimulated cells after 24 hours. **f.** Cell-rounding in hLH-treated cells after 24 hours. LHCGR is labeled in red (Tritc), the cytoplasmic marker ERK1/2 in green (Fitch) and cell nuclei marker (DAPI) in blue. The merging of the three images is in the lower right plate of each panel. Images are from one experiment and are representative of three independent experiments with similar results. White arrows indicated LHCGR; Scale bar: 10 µm. Western blot control for the anti-LHCGR antibody and non-permeabilized cells control for immunofluorescence staining were also included (Fig. S5).

We therefore performed long-term stimulation experiments in which day 6 hGLC were exposed to constant ED_50_ doses of r-hLH (500 pM) or r-hCG (100 pM) for up to 36 hours. As shown in Fig. 2, continuous exposure to r-hLH or r-hCG resulted in repetitive, cyclic rises and falls of intracellular cAMP with peaks approximately every 4–5 hours, while cAMP remained substantially constant in the unstimulated control cells. Moreover, cAMP concentrations during the peaks were often significantly higher upon r-hCG stimulation, in spite of the five-fold lower molar concentrations used, for the first 36 hours (Fig. 3). Intracellular cAMP response tended to extinguish after 30 hours for both r-hLH and r-hCG stimulations. The fluctuations in cellular cAMP response to constant r-hLH or r-hCG doses resulted in progressive, linear accumulation of progesterone in the supernatant, which increased 45-times, from about 200 ng/ml to about 9000 ng/ml, by 36 hours (Fig. 2) for both r-hLH and r-hCG stimulations in spite of overall lower cAMP level stimulated by r-hLH. r-hLH and r-hCG concentrations in the supernatant (measured by an immunometric assay) and cell viability (measured by MTT assay) remained constant during the entire stimulation period (data not shown). The fluctuations in intracellular cAMP were accompanied a constant increase of extracellular cAMP measured in the culture medium (Fig. 2D), suggesting the existence of a mechanism regulating the efflux of the second messenger outside the cell membrane of viable hGLC cells, according to previous observation in a wide variety of cell types [34]. The mechanism of the cyclic hGLC response to constant r-hLH/r-hCG exposure requires a set of *ad hoc* experiments and was not further explored in this setting. However, immunofluorescence staining revealed membrane localization of the LHCGR in unstimulated hGLC at each time-point, while the receptor was visible at the membrane after one hour of stimulation by r-hLH or r-hCG but prevalently intracellular after 15 and 24 hours, suggesting possible internalization (Fig. 4). In addition, stimulated cells tended to assume a typical rounded shape with time, a phenomenon previously described in granulosa cells exposed to FSH [35].

### Acute effects of hLH and hCG on the ERK1/2 and AKT pathways in hGLC

**Figure 5:**
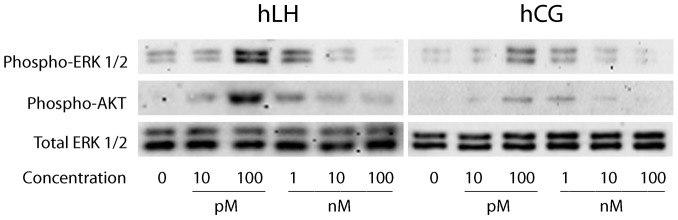
Dose-response experiment evaluating the maximal phospho-ERK1/2 and phospho-AKT activation in hGLC by Western blotting. The cells were stimulated for 15 minutes by different r-hLH or r-hCG doses and the phospho-ERK1/2 and phospho-AKT signals were normalized for total ERK. The image is representative of 3 independent experiments.

We next examined the activation of the ERK1/2- and AKT-pathways in hGLC. First, we performed dose-response experiments in which phospho-ERK1/2 and phospho-AKT were detected by Western blotting of protein extracts obtained from day 6 hGLC stimulated for 15 minutes with different doses of r-hCG or r-hLH in the pM-nM range. As shown in Fig. 5, the highest activation of both ERK1/2 and AKT was reached with 100 pM of either gonadotropin. Interestingly, r-hLH appeared more effective than r-hCG at all doses tested. Differently from what was observed for cAMP, an equipotent (ED_50_) dose of the two gonadotropins on ERK1/2 and AKT activation could not be determined. Therefore, the maximally stimulating 100 pM dose was used for both r-hLH and r-hCG in further experiments.

As shown for cAMP dose-response experiments (Fig. S2), a comparison between recombinant and extractive gonadotropins was performed in hGL5/LHCGR cells by Western blot, to assess whether the carbohydrate structure related to recombinant gonadotropins could affect the phosphorylation rate of ERK1/2 and AKT (Fig. S6). The results obtained were similar, indicating that recombinant and extractive gonadotropins lead to essentially the same effects.

**Figure 6:**
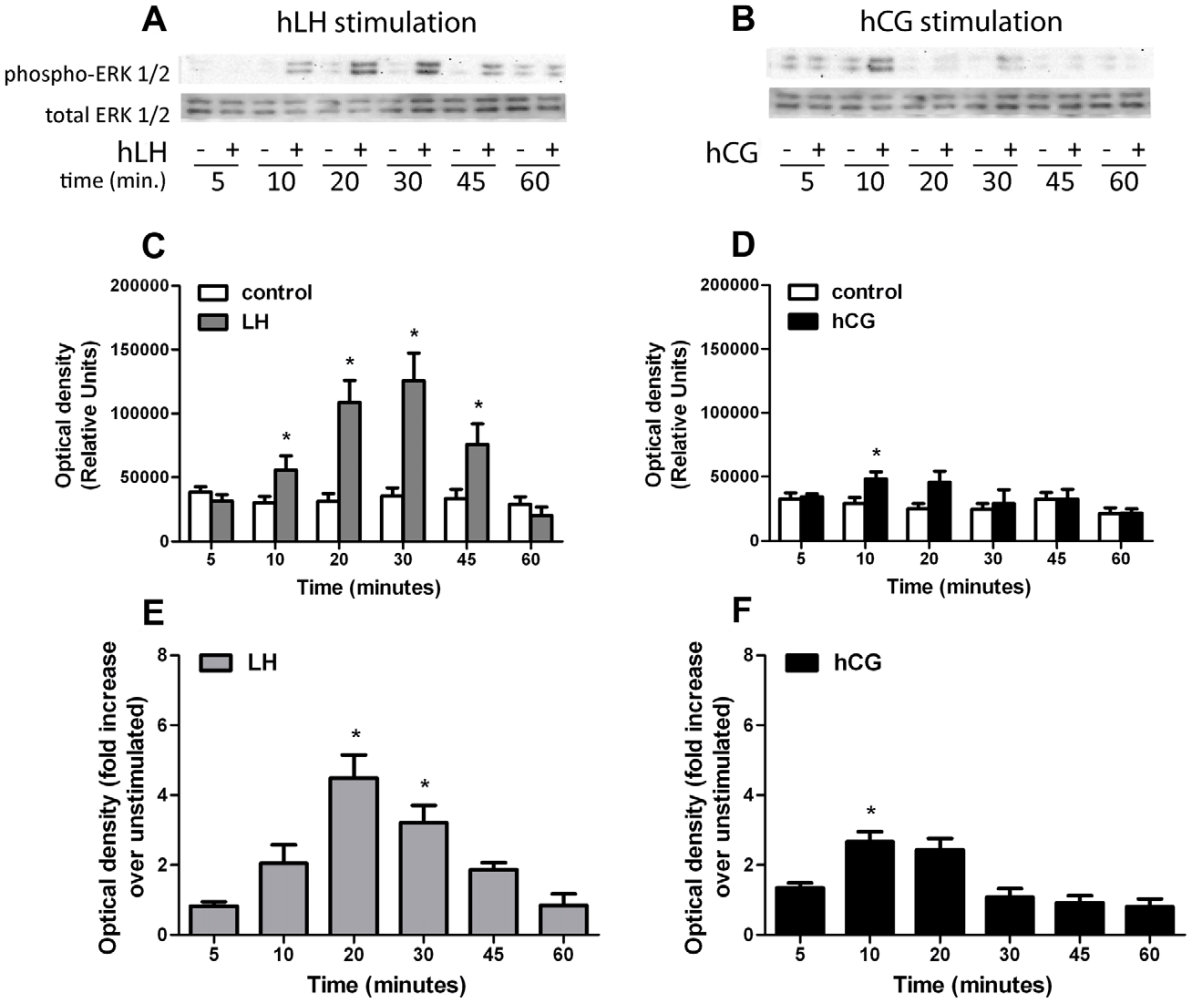
Time-course analysis of the phospho-ERK1/2 activation in hGLC under 100 pM hLH or 100 pM hCG stimulation. **a.** Comparison of phospho-ERK1/2 activation in hLH-stimulated *vs* unstimulated (control) at different time-points, by Western blotting (image is representative of 4 independent experiments; total ERK as normalizer). **b.** Comparison of phospho-ERK1/2 activation in hCG-stimulated *vs* unstimulated (control) at different time-points, by Western blotting (image is representative of 4 independent experiments; total ERK as normalizer). **c, d.** Relative semi-quantification of the optical density representing phospho-ERK1/2 activation (shown in figs. 6A,B) stimulated by (**c**) hLH or (**d**) hCG, compared to unstimulated (Mean±SEM; n = 4; * = significant *vs* unstimulated; *t-test*; *p*<0.05). **e.** Normalization of the phospho-ERK1/2 signals measured in hLH-stimulated samples (represented as relative units in Fig. 6C) over each unstimulated (Mean±SEM; n = 4; * = significant *vs* unstimulated; *two-way analysis of variance*; *p*<0.05). **f.** Normalization of the phospho-ERK1/2 signals measured in hCG-stimulated samples (represented as relative units in Fig. 6D) over each unstimulated (Mean±SEM; n = 4; * = significant *vs* unstimulated; *two-way analysis of variance*; *p*<0.05).

**Figure 7:**
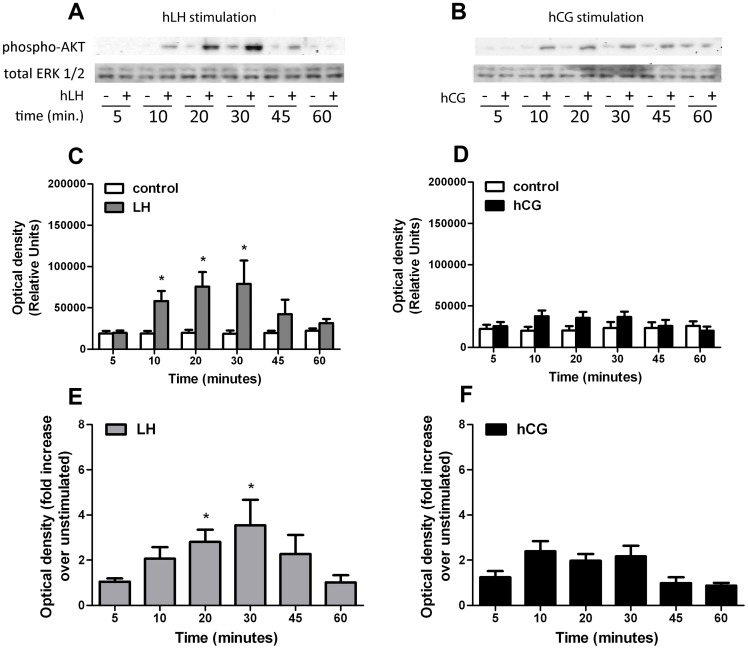
Time-course analysis of the phospho-AKT activation in hGLC under 100 pM hLH or 100 pM hCG stimulation. **a.** Comparison of phospho-AKT activation in hLH-stimulated *vs* unstimulated (control) at different time-points, by Western blotting (image is representative of 4 independent experiments; total ERK as normalizer). **b.** Comparison of phospho-AKT activation in hCG-stimulated *vs* unstimulated (control) at different time-points, by Western blotting (image is representative of 4 independent experiments; total ERK as normalizer). **c, d.** Relative semi-quantification of the optical density representing phospho-AKT activation (shown in figs. 7A,B) stimulated by (**C**) hLH or (**D**) hCG, compared to unstimulated (Mean±SEM; n = 4; * = significant *vs* unstimulated; *t-test*; *p*<0.05). **e.** Normalization of the phospho-AKT signals measured in hLH-stimulated samples (represented as relative units in Fig. 7C) over each unstimulated (Mean±SEM; n = 4; * = significant *vs* unstimulated; *two-way analysis of variance*; *p*<0.05). **f.** Normalization of the phospho-AKT signals measured in hCG-stimulated samples (represented as relative units in Fig. 7D) over each unstimulated (Mean±SEM; n = 4; * = significant *vs* unstimulated; *two-way analysis of variance*; *p*<0.05).

The kinetics of ERK1/2 and AKT activation were studied in time-course experiments using day 6 hGLC in a time range of 5–60 min. Phospho-ERK1/2 and phospho-AKT signals were detected by Western blot and semiquantified by image analysis. Stimulation with 100 pM of r-hLH resulted in a strong, significant activation of ERK1/2 between 10 and 45 minutes, while 100 pM of r-hCG induced a much weaker and short-lived stimulation, reaching significance only at 10 min (Fig. 6). Similar effects were observed for phospho-AKT. Here again r-hLH (100 pM) provoked a significant increase in phospho-AKT between 10 to 30 minutes, while the r-hCG (100 pM) stimulation appeared to be minimal and did not reach statistical significance at any time point (Fig. 7). The ERK1/2 and AKT activation tended to extinguish within 60 minutes for both r-hLH and r-hCG stimulations.

These data demonstrate that, in hGLC, r-hLH acutely activates ERK1/2 and AKT, while r-hCG action is weaker, less sustained and significant only on the ERK1/2 pathway.

### Effects of early blockade of the ERK1/2 or AKT pathway on hLH- and hCG-mediated gene expression

**Figure 8:**
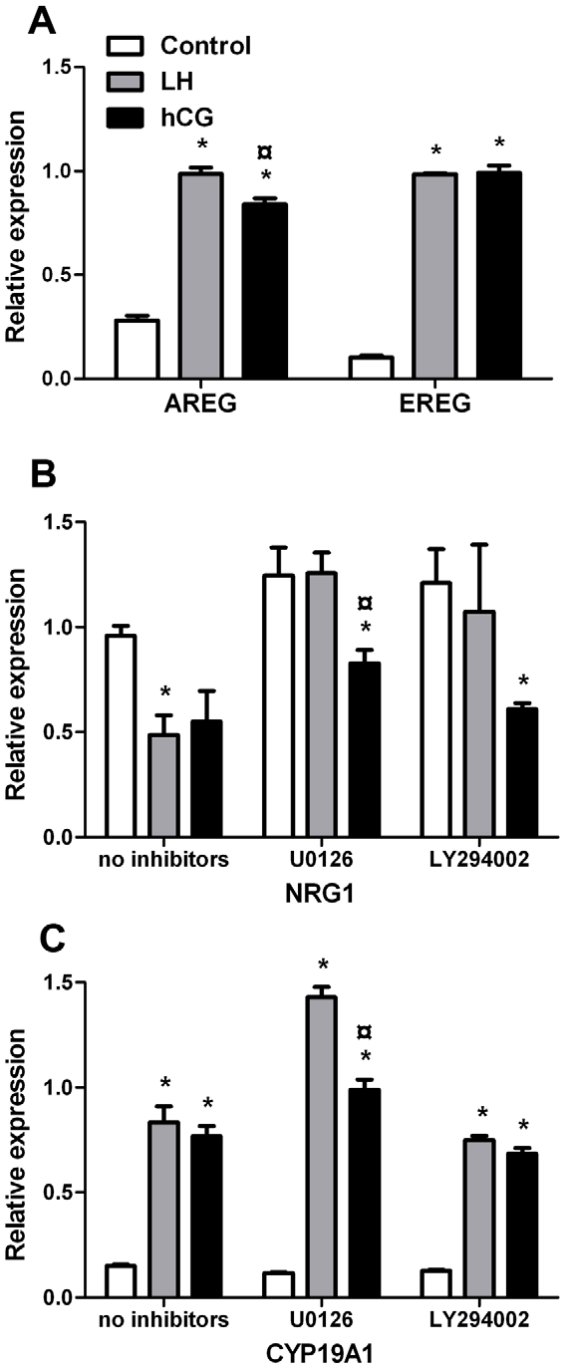
Evaluation of the gene expression induced in hGLC by hLH or hCG stimulation after 12 hours, performed by real-time PCR. Specific ERK1/2- or AKT-pathways inhibitor (U0126 and LY294002 respectively) were also used where indicated. **a.** Increase in the gene expression of the EGF-like factors amphiregulin (*AREG*) and epiregulin (*EREG*) induced by LH or hCG. **b.** Effects of the hLH or hCG stimulation and of the ERK1/2- or AKT pathway early inhibition on neuregulin 1 (*NRG1*) gene expression. **c.** Effects of the hLH or hCG stimulation and of the ERK1/2- or AKT pathway early inhibition on *NRG1* gene expression. In each treatments, *RPS7* gene expression was used as normalizer (mean±SEM; n = 4; * = significant *vs* control; ° = significant *vs* hLH-stimulated; *t-test*; *p*<0.05).

Given the different effect of r-hLH and r-hCG on acute ERK and AKT activation, we assessed whether the selective blockade of these pathways could affect the expression of genes known to be under hLH and/or hCG control. Day 6 hGLC where exposed to the specific inhibitors U0126 or LY294002 for one hour and then stimulated with either r-hLH or r-hCG (100 pM). After 12 hours gene expression of the EGF-like factors amphiregulin (*AREG*), epiregulin (*EREG*) and neuregulin 1 (*NRG1*) and of *CYP19A1* (aromatase) was quantified by real-time RT-PCR. r-hLH or r-hCG stimulation resulted in significant stimulation of the expression of *AREG* and *EREG* and inhibition of *NRG1* (Fig. 8A and 8B) and significant stimulation of aromatase (Fig. 8C). In addition, r-hLH was significantly more potent than r-hCG on *AREG*.

Pre-incubation of hGLC with U0126 or LY294002 resulted in sustained inhibition of ERK1/2 and AKT phosphorylation, respectively (Fig. S7). The r-hLH- and r-hCG-stimulated expression of *AREG* and *EREG* was not changed by U0126 or LY294002 (data not shown), while inhibition of the ERK or AKT pathway exerted significant effects on *NRG1* expression r-hLH- but not r-hCG-mediated (Fig. 8B). In fact, both U0126 and LY294002 prevented the decrease of *NRG1* expression induced by r-hLH, indicating that both ERK- and AKT-pathways are involved in the inhibition of *NRG*1 expression controlled by r-hLH. Conversely, neither U0126 nor LY294002 had any effect on r-hCG-inhibited *NRG1* expression, suggesting that the early activation of these pathways is not involved in r-hCG-mediated action on neuregulin 1, a result consistent with the very weak (or lacking) activation of these pathways by r-hCG as demonstrated in Fig. 6 and Fig. 7.

As shown in Fig. 8C, the r-hLH- and r-hCG-stimulated gene expression of *CYP19A1* was not affected by LY294002, while U0126 significantly and specifically increased only r-hLH-stimulated expression. These results demonstrate that the early activation of the ERK1/2 pathway is involved in hLH- but not in hCG action on *CYP19A1* expression while AKT activation plays no role.

## Discussion

We systematically analyzed whether hLH and hCG are equivalent *in vitro* in terms of biopotency, kinetics of response and molecular effects. It is traditionally believed that hLH and hCG are biologically equivalent since they act *via* the same receptor, for which both molecules are assumed to have the same binding affinity. hCG has long been the preferred hormone both in clinical practice and in *in vitro* experiments because, before the advent of recombinant gonadotropins, it could be obtained easily by urinary extraction and owing to its much longer half-life. However, hLH and not hCG is the physiological hormone in non-pregnant women and the evolutionary reason for the presence of hCG in primates has not been clearly established. The vast majority of our current knowledge about *in vitro* hLH action on Leydig and granulosa/theca cells was obtained using hCG. Our experiments challenge the concept that hLH and hCG have the same biopotency and bioactivity.

### Acute cAMP response

Our results demonstrate that, *in vitro*, in the presence of a standardized receptor milieau and at maximal stimulation, hCG is about 5-times more biopotent than equimolar concentrations of hLH in terms of cAMP production. Previous *in vitro* [36] and *in vivo* [37]–[39] studies did not pick up differences between the effects of hCG and hLH, probably because they were based on gonadotropins not quantified in exact molar terms but in “units” calibrated against a third preparation, e.g. the standard International Standard WHO 80/522 [18], or using *in vivo* bioassay (rat seminal vesicle weight gain assay). In the latter system, the biopotency ratio between the two molecules has been evaluated as 1∶6 [40] meaning that, when amounts of hLH and hCG equipotent *in vivo* are used *in vitro*, the molar ratio favours hLH, thereby masking the higher activity of hCG at the receptor level, which becomes evident when the two gonadotropins are used *in vitro* at equimolar concentrations. Essentially the same results were observed when hGLC were used to assess hLH and hCG ED_50_ (Fig. S3).

The equipotent doses of hLH and hCG were determined by measuring total cAMP over 3 hours (Fig. 1A) and were confirmed by progesterone production in hGLC, which reached equal levels upon hLH or hCG stimulation (Fig. 2). When half-maximal, ED_50_ concentrations were used in time course-experiments, intracellular cAMP accumulation resulted about 6-times faster in response to hLH than hCG (Fig. 1B). Whether the rate of cAMP increase depends on the levels of receptor occupation, on receptor dimerization, or on G protein coupling is still unknown. Binding studies performed formerly with the same cell line [18] showed that hLH was significantly less potent than hCG in displacing [^125^I]hCG, suggesting different binding kinetics. However, this is not sufficient to establish a difference in receptor affinity for the two hormones, since the same studies have not been performed in parallel using [^125^I]hLH. Binding studies with [^125^I]hCG and [^125^I]hLH would be necessary to assess whether the two hormones have different affinity/dissociation features. Future experiments should investigate which residues on the LHCGR, possibly in exon 10, are involved in distinguishing between the two hormones, whether both hLH and hCG induce receptor dimerization, and whether the same G proteins are activated by the two hormones.

Since hCG is a primate-specific hormone naturally produced mainly during pregnancy, it has been speculated that its occurrence in the evolution of primates might be related to the complexity of hemochorial placentation [41], [42]. At pregnancy onset the rescue of the corpus luteum is ensured by very low amounts of hCG produced by the early embryo and pituitary hLH is not sufficient for this task. Our cAMP *in vitro* data suggest that, in an *in vivo*, “physiological” setting, sustained progesterone production could take advantage from the higher activity of hCG, compared to hLH, at the receptor level, in addition to its longer *in vivo* half-life.

### Comparison between recombinant and extractive gonadotropins

The use of recombinant hLH/hCG provides the unique possibility of comparing effects of both hormones at equimolar concentrations. Nevertheless, recombinant and extractive gonadotropins could differ in extent and type of glycosylation. To assess whether the possibly different tertiary structure of recombinant and extractive gonadotropins affect the cell signaling, a comparison between both type of gonadotropins was performed in different cell models, showing similar results and indicating that recombinant and extractive gonadotropins result in essentially comparable effects (Fig. S2 and Fig. S6).

hLH is secreted as multiple forms by the pituitary, as suggested by the structural and functional heterogeneity of at least 20 different hLH isoforms with various sialic acid content [43], [44], depending from the purification method used and the source, pituitary or urinary [45].

hCG is also a heterogenic molecule. It is produced from several cell types in at least five different forms, (e.g. from cytotrophoblast, syncytiotrophoblast, pituitary, tumor tissue, etc.) each having the same amino acid sequence but showing different function depending on the type and heaviness of glycosylation due to O-linked and N-linked sugars [46], [47]. Recombinant hLH and hCG are well described [48]–[50] and they are produced industrially with the goal of achieving highly consistent molecules by using a consistent and standardized manufacturing process. However, there is no extensive physicochemical comparative characterization of recombinant and natural, extractive hLH and hCG molecules, due to the lack of a highly purified reference preparation and to the different analytical methods used [51].

Using chromatographic procedures the retention time of the natural and recombinant hLH and hCG molecules are similar, demonstrating similar hydrophobicity of the alpha and beta subunits [45]. The only available comparative data between the commercial r-hLH and r-hCG versus the natural forms are referred to pharmacokinetics and pharmacodynamic studies. Both recombinant hLH and extractive hLH were eliminated with a terminal half-life of few hours. The pharmacokinetics of recombinant human LH are dose-dependent and similar to those of extractive human LH [52]. The same holds true for hCG, since r-hCG and ex-hCG produced bioequivalent pharmacodynamic responses consistent with the natural physiology of hCG [53].

### Chronic cAMP and progesterone response

The experiments with hGLC revealed for the first time that intracellular cAMP response to constant, chronic stimulation by hLH or hCG is cyclic, with a period of about 4–5 hours. The hGLC cell model is naturally expressing the LHCGR and it can be presumed that the receptor undergoes a kind of “physiological” regulation upon stimulation. Differently, LHCGR gene transcription is regulated by the CMV promoter in COS-7/LHCGR and some discrepancies between hGLC could exist, in terms of intracellular cAMP regulation. This is the main reason why the hGLC cell model was used in long-term time-course experiments. Interestingly, although equipotent doses of hLH and hCG were used, after the first two-three hours of stimulation cAMP production was significantly higher in response to hCG than to hLH at a few time points. The consequence of the intermittent, intracellular cAMP production was a progressive, massive secretion of progesterone (a terminal product of steroidogenesis in granulosa-lutein cells) in the supernatant. Therefore, constant exposure to hLH or hCG at concentrations close to those circulating in vivo (as assessed by measuring hLH and hCG by ELISA in the supernatant) results in the amplification of the steroidogenic response over time. This is consistent with the clinical experience that gonadotropin administration does not need to be pulsatile and with experimental studies in mice [54], in which continuous and pulsatile infusions of hLH have identical steroidogenic effects in rats passively immunized against gonadotropin-releasing hormone. Therefore, hLH/hCG-responsive cells, such as hGLC, have developed a mechanism of cyclic refractoriness of the LHCGR to the chronic, non-pulsatile and increasing stimulation by hCG occurring in pregnancy, resulting in a progressive increase of progesterone production. Conversely, pituitary hLH secretion in non-pregnant women is physiologically pulsatile and at much lower concentrations compared to hCG during pregnancy. We speculate that the evolutionary reason why the two hormones result in these different cAMP production features could be found in the main role of cAMP plays in sustaining steroidogenesis as required in pregnancy [55], while LH has higher activity during folliculogenesis, when the cell cycle regulators AKT and ERK play a crucial role [56], [57]. The molecular mechanism regulating the fluctuations in intracellular cAMP response remains to be extensively studied. The presence of cAMP in the culture medium (Fig. 2D) strongly suggests the existence of a mechanism regulating the efflux of the second messenger outside the cell membrane of viable hGLC cells, according to previous observations in a wide variety of cell types [34]. In this regard, in primary rat skeletal muscle cultures the loss of intracellular cAMP from peak levels, induced by activation of adenylyl cyclase, was followed by an increase of extracellular cAMP. This effect was not dependent on phosphodiesterases activation, since it was obtained in the presence of IBMX [58]. Moreover, hGLC are able to downregulate this receptor after *in vivo* or *in vitro* stimulation with gonadotropins [28]. Internalization of the ligand-receptor complex, downregulation of the mRNA transcript [31] and phosphorylation of serine or threonine residues on the receptor molecules [1], [28] could be involved. In addition, receptor dimerization, a feature common to all the G-protein coupled receptors (GPCRs) [2], might play a role in the activation of different signalling pathways [59], resulting in the cyclic waving of intracellular cAMP. Finally, post-endocytotic trafficking of the hormone-receptor complex, which has already been extensively studied for the FSH receptor (FSHR) [61], the thyroid-stimulating hormone receptor (TSHR) [61], [62] and others GPCRs [63] could be involved.

Interestingly, hLH and hCG were equipotent in terms of progesterone production in spite of overall lower cAMP levels when stimulated by hLH. Progesterone production in preovulatory granulosa cells depends on the cAMP/PKA-pathway [64] but other signalling pathways could be involved [57], e.g. through molecules of the EGF family such as neuregulin 1 and amphiregulin [22], [65], modulated by ERK1/2 and AKT [66].

### Acute activation of the ERK1/2 and AKT pathways and gene expression

Our data show for the first time in hGLC, that the acute effect of maximally stimulating doses of hCG on ERK1/2 phosphorylation is low when compared to the powerful effect of hLH. These data were corroborated by the experiments investigating the consequences of selective inhibition of early ERK1/2 and AKT activation on the expression of selected hLH- and hCG-dependent genes. Therefore, while hCG is more potent than LH on the PKA pathway, hLH is more potent than hCG on the ERK1/2 and AKT pathways. Interestingly, similar findings were very recently reported in goat ovarian granulosa cells after prolonged treatment with supra physiological doses of hLH and hCG [67]. In these experiments the role of hLH and hCG was investigated in relation to tumorigenesis and it was shown that hLH promoted growth and proliferation in caprine ovarian granulosa cells, while hCG was more active on stimulating cAMP levels and decreased the rate of proliferation.

The functional role of the cAMP, ERK and AKT signalling pathways in fertility is actively investigated both in humans [22] and animal models [57], [68], [69], revealing that hLH/hCG stimulation of the same receptor results in activation of different, complex signal transduction pathways and molecules [70]–[72]. Although recent findings attribute to cAMP the ability to mediate multiple and opposite effects [73], the *in vitro* activation of cAMP-pathway by gonadotropins is traditionally associated to structural changes, consisting in cell-rounding [19], [74], apoptotic events [74]–[76] and to prevention of meiosis resumption in the oocyte [77]. In contrast, gonadotropin-dependent activation of anti-apoptotic pathways [22], [78] and proliferative effects [79] seems to be mediated by ERK1/2 and AKT, and reduction of ERK1/2 signalling activates apoptotic signals [80]. Granulosa cell death in vitro can be induced by the specific AKT inhibitor LY294006 and is completely blocked by hLH co-treatment [78]. Taken together, these results indicate that hCG and hLH action on the regulation of cell cycle and apoptosis in granulosa cell might be divergent and/or dependent on which signal transduction pathway is activated. This is particularly relevant in determining the cell fate during folliculogenesis, when the activation of different signal transduction pathways mediates a delicate balance between pro- and anti-apoptotic signals [80].

The continuous stimulation of granulosa cells by hLH/hCG over 12 hours resulted in the increase of *AREG*, *EREG* and *CYP19A1* gene expression and in the reduction of *NRG1* gene expression. The EGF-like factors *AREG*, *EREG* and *NRG1* are well known target genes of hLH/hCG action [66], [81], [82]. These molecules may play a role in the ovulatory process and oocyte maturation [66], [83] exerted *via* both ERK- and AKT-pathways activation [22],[71]. By using the U0126 or LY294002 inhibitors, we confirmed the different involvement of the ERK1/2 and AKT pathways on gene expression in dependence of hLH or hCG. In accordance with the lower AKT activation by hCG *versus* hLH, the inhibition of this pathway by LY294002 did not result in any relief of the hCG-dependent inhibition of *NRG1* expression, while the inhibitory effect mediated by hLH was prevented by blocking AKT. Conversely, only the decrease of *NRG1* gene expression induced by hLH was counteracted by inhibition of ERK1/2, indicating again the relevance of this pathway for hLH- but not for hCG action. Finally we investigated the expression of *CYP19A1* gene, an early-response target of hLH and hCG both in human [25], [84] and in mice [85]. Our data demonstrated a further increase of *CYP19A1* gene expression in U0126 treated cells under hLH- but not hCG stimulation. In contrast, AKT-pathway blockade did not affect *CYP19A1* gene expression. This suggests that the ERK-pathway exerts a negative control on aromatase expression mediated by hLH but not hCG, pinpointing another relevant difference in the mechanism of action of the two gonadotropins in hGLC.

## Conclusions

The present work provides novel insights in the downstream signaling of the LHCGR in response to hLH and hCG stimulation. For the first time we provide evidence that equimolar concentrations of hLH and hCG result in a higher *in vitro* activity of hCG versus hLH in terms of cAMP. Moreover, in hGLC the LHCGR is able to differentiate the *in vitro* hLH and hCG action at the molecular level, affecting the kinetics of cAMP production and ERK1/2- and AKT-pathway activation. Different signal transduction pathways were also differentially used to reach an equal transcriptional level of target genes by both gonadotropins, but how LHCGR modulates the activation of different signalling pathways depending on the ligand remains to be clarified. Our study was so far limited at assessing the acute effects of hLH and hCG and future experiments should be dedicated to the events following the early activation of the various pathways and their interaction, resulting in the final, full biological effects. Finally, while the *in vivo* effects of the differential activation of the various pathways remain to be studied in detail, the nonequivalence of hLH and hCG deserves consideration in the development of future therapeutic strategies [86].

## Supporting Information

Figure S1 **Recovery of hGLC response to hLH and hCG, over 0–6 days from** ***in vivo*** **pick-up.** Total cAMP measured on the (**a**) third and (**b**) sixth day of culture are shown.
(TIF)

Figure S2 **Comparison between recombinant and extractive gonadotropins effects on total cAMP production.** **a.** Dose-response experiment with r-hLH and r-hCG *versus* ex-hLH and ex-hCG in COS7/LHCGR, in the presence of 500 µM IBMX. Total cAMP was measured after 3 hours. One representative experiment is shown. **b.** The experiment shown in panel “**a**” has been repeated using hGL5/LHCGR, in the presence of 500 µM IBMX. Total cAMP was measured after 3 hours. One representative experiment is shown.
(TIF)

Figure S3 **Dose-response and time-course experiments in hGLC.** **a.** Dose-response experiment with r-hLH and r-hCG in hGLC in the presence of 500 µM IBMX. Total cAMP was measured after 3 hours. One of three independent experiments is shown. **b.** Time-course experiment performed by continuous incubation of hGLC for different time-points in the presence of 500 µM IBMX and gonadotropins at ED_50_ doses (500 pM r-hLH; 100 pM r-hCG). Intracellular cAMP was measured. One of three independent experiments is shown in absolute levels.
(TIF)

Figure S4 Intracellular cAMP production over 6 hours in hGLC stimulated by hLH (500 pM) or hCG (100 pM). Each value was normalized *vs* unstimulated. One representative experiment of two is shown.
(TIF)

Figure S5 **Control samples of immunofluorescence analysis.** *Non*-permeabilized cells control of LHCGR sequestration from cell surface in hLH-treated hGLC, after 15 hours. **a.** Unstimulated cells. **b.** hLH-treated hGLC. LHCGR is labeled in red (Tritc), the cytoplasmic marker ERK1/2 in green (Fitch) and cell nuclei marker (DAPI) in blue. The merging of the three images is in the lower right plate of each panel. Images are from one experiment and are representative of three independent experiments with similar results. **c.** Western blot control for anti-LHCGR antibody performed on 1) COS7/LHCGR cell lysates; 2) hGL5/LHCGR cell lysates; 3) Untrasfected hGL5 cell lysates (negative control); 4) and 5) hGLC cell lysates from two different donors.
(TIF)

Figure S6 **Comparison between recombinant and extractive gonadotropin effect on ERK1/2 and AKT phosphorylation.** Dose-response experiment evaluating the maximal phospho-ERK1/2 and phospho-AKT activation in hGL5/LHCGR by Western blotting. The cells were stimulated for 15 minutes by different recombinant or extractive hLH or hCG doses and the phospho-ERK1/2 and phospho-AKT signals were normalized for total ERK. One representative experiment is shown.
(TIF)

Figure S7 Western blot analysis of phospho ERK1/2 (**a**) and phospho AKT (**b**) and efficacy of the relative inhibitors U0126 and LY294002 in hGLC stimulated for 15 with hLH or hCG.
(TIF)

Methods S1 **Supplementary Methods**
(DOCX)
