# Supplementary figures and images for: LH and hCG Action on the Same Receptor Results in Quantitatively and Qualitatively Different Intracellular Signalling
Source: PLoS One. 2012 Oct 5;7(10):e46682. doi: 10.1371/journal.pone.0046682 (PMC3465272; doi:10.1371/journal.pone.0046682)

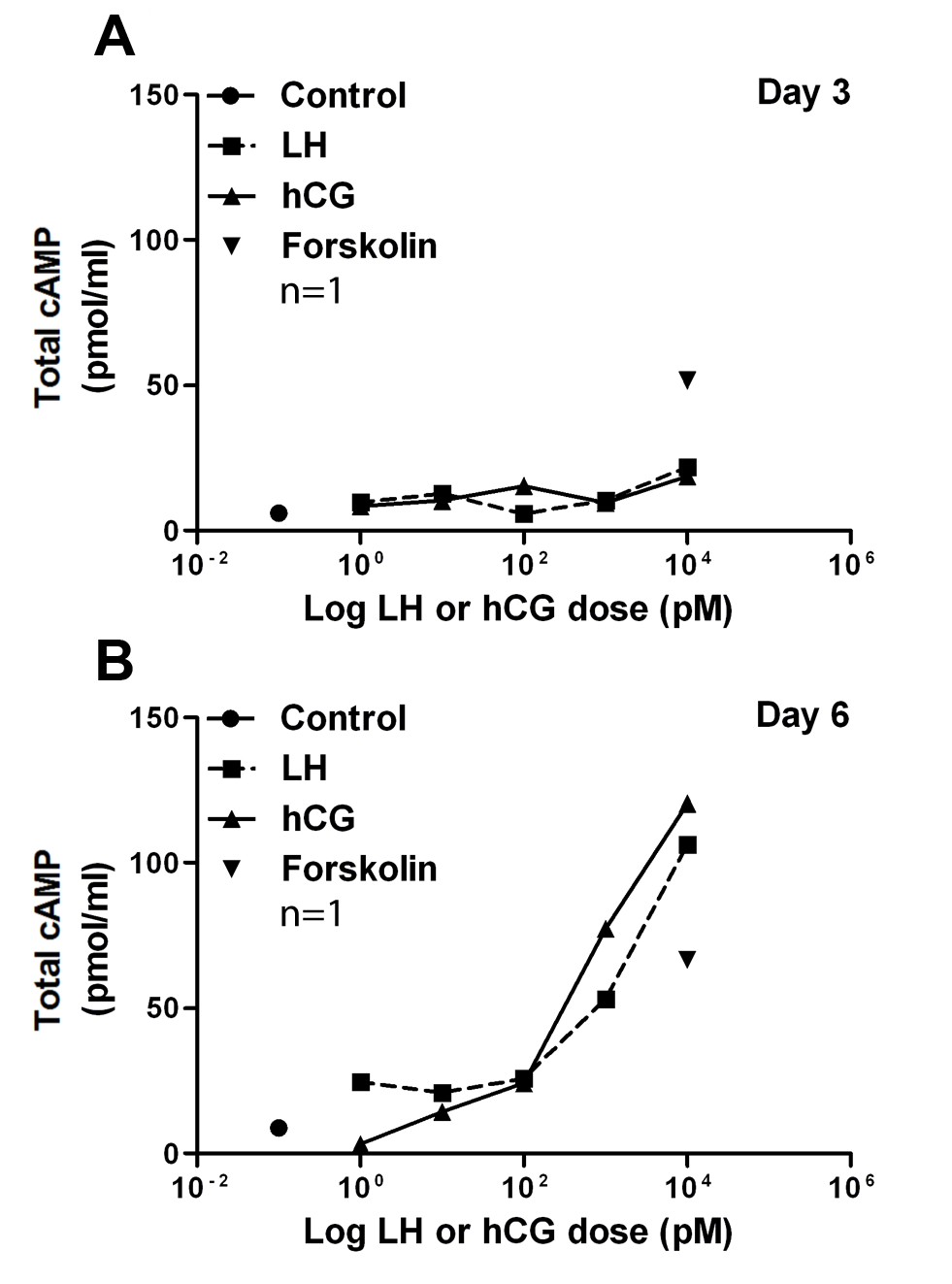

Supplement: Figure S1 — Recovery of hGLC response to hLH and hCG, over 0–6 days from in vivo pick-up. Total cAMP measured on the (a) third and (b) sixth day of culture are shown. (TIF) [file pone.0046682.s001.tif]

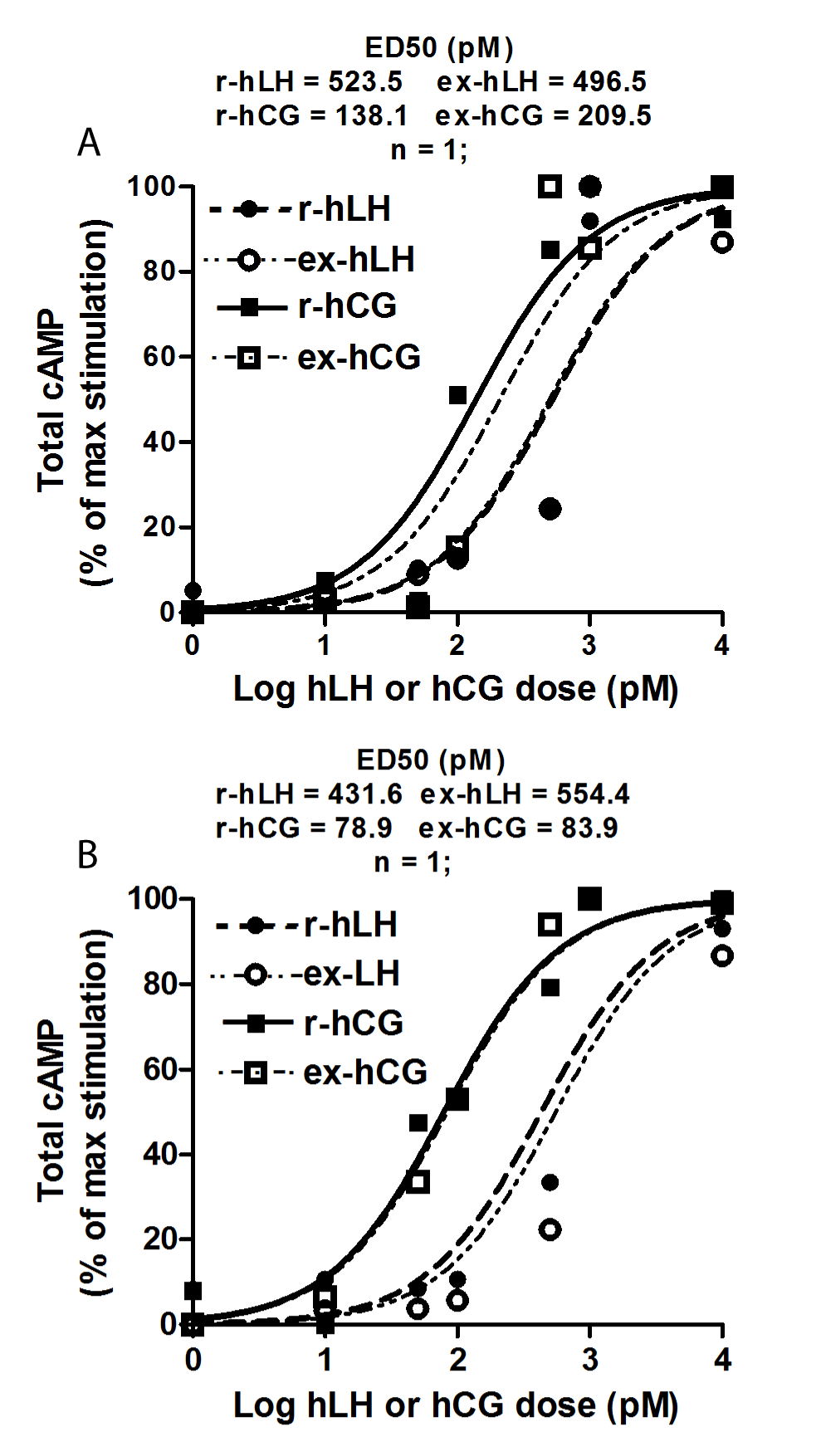

Supplement: Figure S2 — Comparison between recombinant and extractive gonadotropins effects on total cAMP production. a. Dose-response experiment with r-hLH and r-hCG versus ex-hLH and ex-hCG in COS7/LHCGR, in the presence of 500 µM IBMX. Total cAMP was measured after 3 hours. One representative experiment is shown. b. The experiment shown in panel “a” has been repeated using hGL5/LHCGR, in the presence of 500 µM IBMX. Total cAMP was measured after 3 hours. One representative experiment is shown. (TIF) [file pone.0046682.s002.tif]

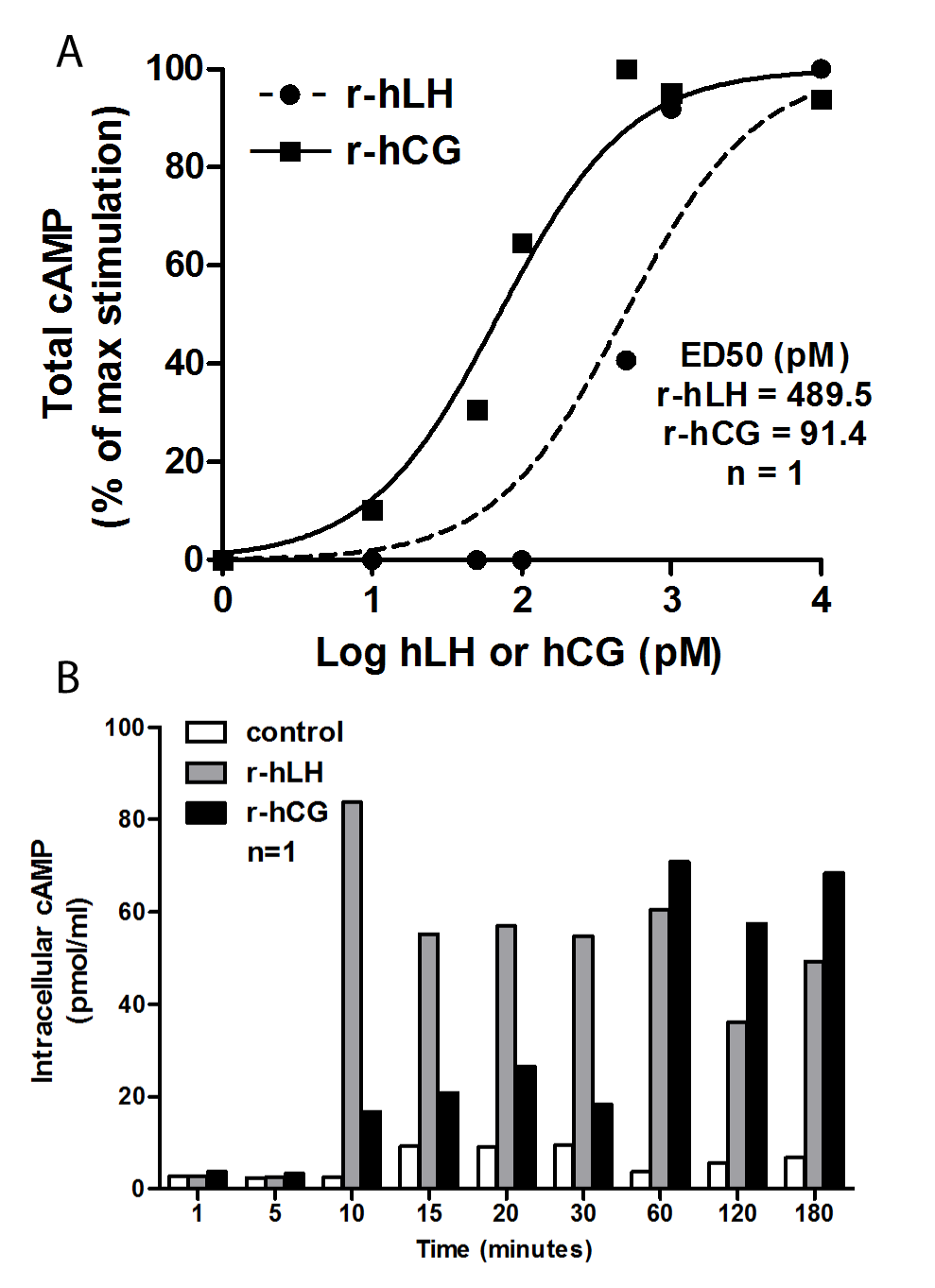

Supplement: Figure S3 — Dose-response and time-course experiments in hGLC. a. Dose-response experiment with r-hLH and r-hCG in hGLC in the presence of 500 µM IBMX. Total cAMP was measured after 3 hours. One of three independent experiments is shown. b. Time-course experiment performed by continuous incubation of hGLC for different time-points in the presence of 500 µM IBMX and gonadotropins at ED50 doses (500 pM r-hLH; 100 pM r-hCG). Intracellular cAMP was measured. One of three independent experiments is shown in absolute levels. (TIF) [file pone.0046682.s003.tif]

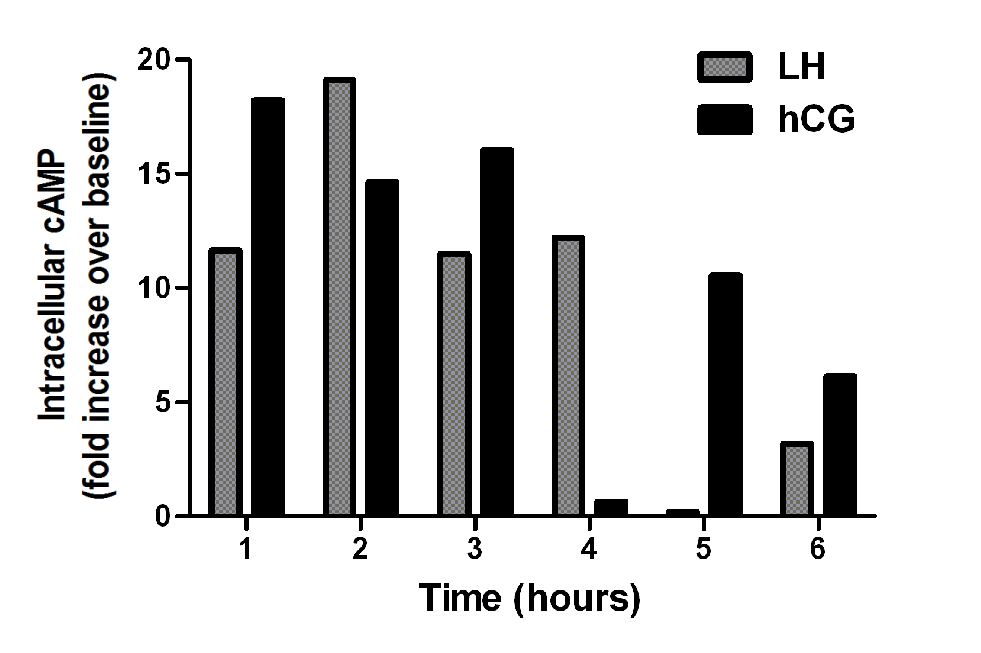

Supplement: Figure S4 — Intracellular cAMP production over 6 hours in hGLC stimulated by hLH (500 pM) or hCG (100 pM). Each value was normalized vs unstimulated. One representative experiment of two is shown. (TIF) [file pone.0046682.s004.tif]

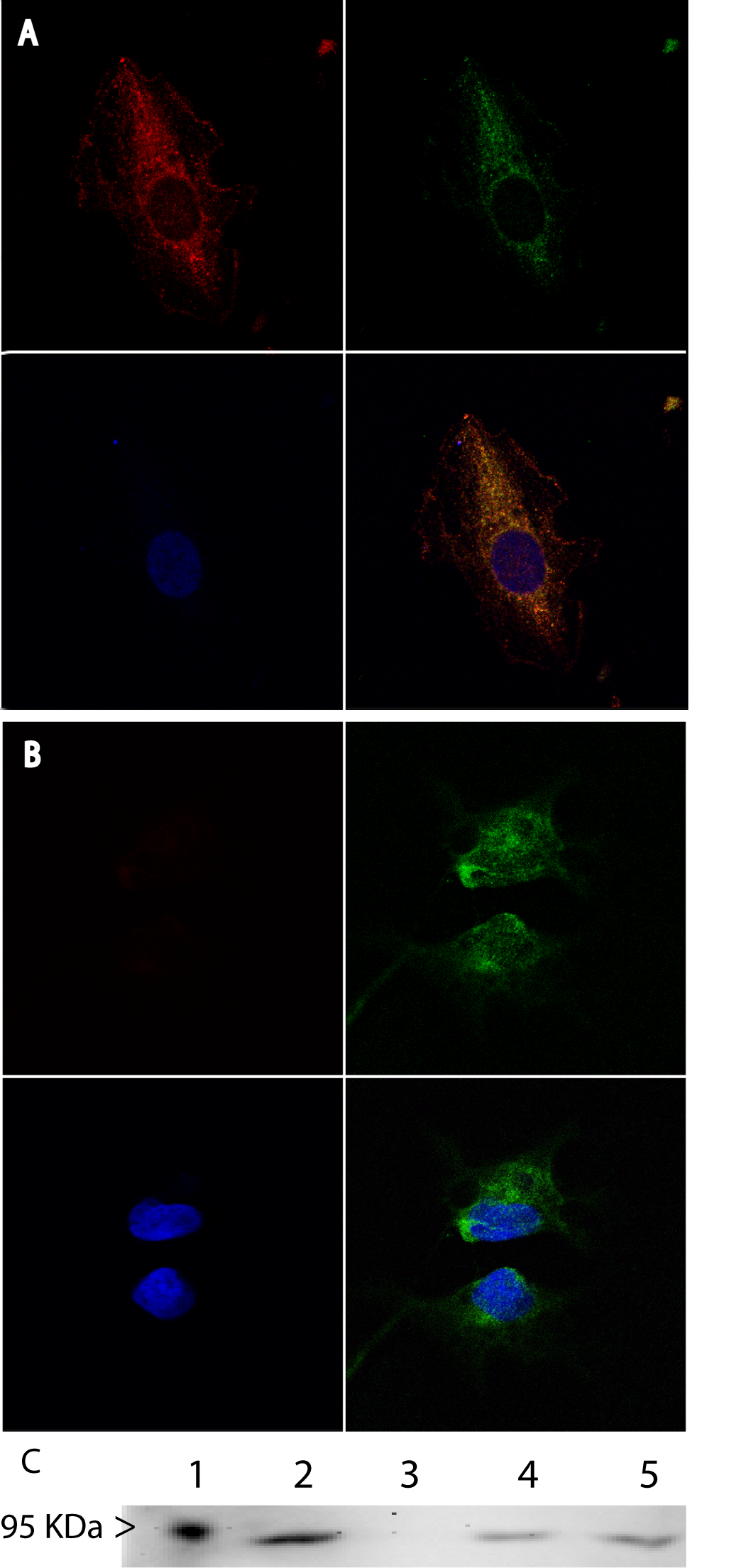

Supplement: Figure S5 — Control samples of immunofluorescence analysis. Non-permeabilized cells control of LHCGR sequestration from cell surface in hLH-treated hGLC, after 15 hours. a. Unstimulated cells. b. hLH-treated hGLC. LHCGR is labeled in red (Tritc), the cytoplasmic marker ERK1/2 in green (Fitch) and cell nuclei marker (DAPI) in blue. The merging of the three images is in the lower right plate of each panel. Images are from one experiment and are representative of three independent experiments with similar results. c. Western blot control for anti-LHCGR antibody performed on 1) COS7/LHCGR cell lysates; 2) hGL5/LHCGR cell lysates; 3) Untrasfected hGL5 cell lysates (negative control); 4) and 5) hGLC cell lysates from two different donors. (TIF) [file pone.0046682.s005.tif]

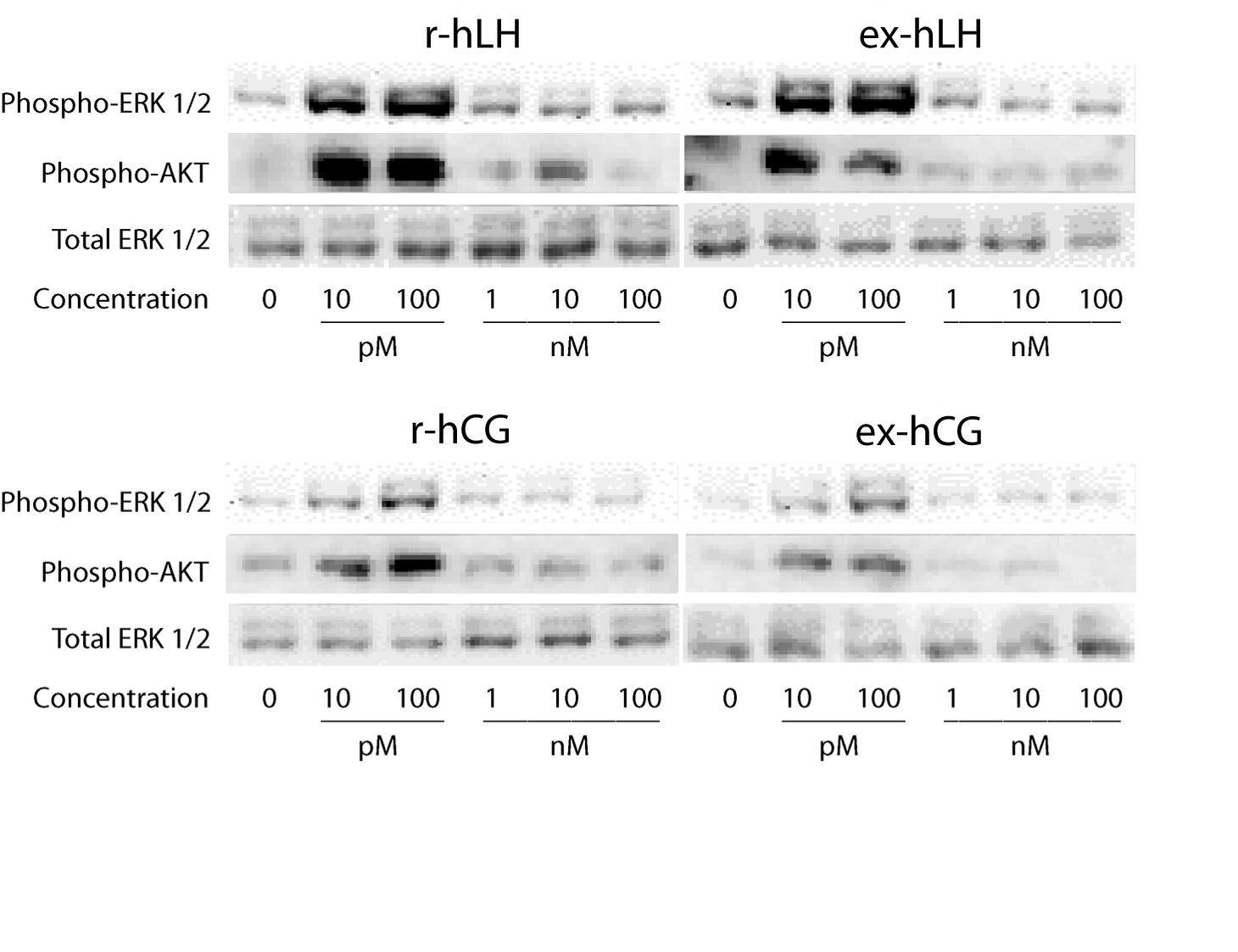

Supplement: Figure S6 — Comparison between recombinant and extractive gonadotropin effect on ERK1/2 and AKT phosphorylation. Dose-response experiment evaluating the maximal phospho-ERK1/2 and phospho-AKT activation in hGL5/LHCGR by Western blotting. The cells were stimulated for 15 minutes by different recombinant or extractive hLH or hCG doses and the phospho-ERK1/2 and phospho-AKT signals were normalized for total ERK. One representative experiment is shown. (TIF) [file pone.0046682.s006.tif]

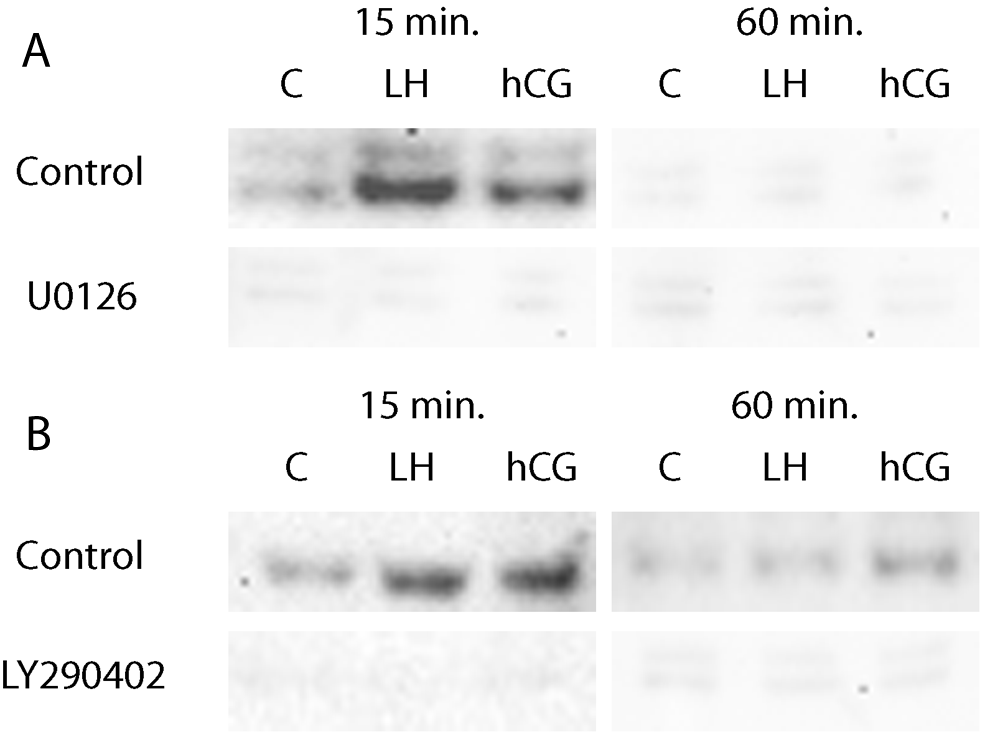

Supplement: Figure S7 — Western blot analysis of phospho ERK1/2 (a) and phospho AKT (b) and efficacy of the relative inhibitors U0126 and LY294002 in hGLC stimulated for 15 with hLH or hCG. (TIF) [file pone.0046682.s007.tif]
