# Supplementary material for: LH and hCG Action on the Same Receptor Results in Quantitatively and Qualitatively Different Intracellular Signalling
Source: PLoS One. 2012 Oct 5;7(10):e46682. doi: 10.1371/journal.pone.0046682 (PMC3465272; doi:10.1371/journal.pone.0046682)
Supplement: Methods S1 — Supplementary Methods (DOCX) [file pone.0046682.s008.docx]

**Supplementary methods**

**Recombinant and extractive gonadotropins.** Highly purified recombinant r-hLH (Luveris) and r-hCG (Ovitrelle) were kindly provided by Merck-Serono S.p.A. (Rome, Italy). Luteinizing hormone extracted from human pituitary (ex-hLH) and chorionic gonadotropin extracted from human pregnancy urine (ex-hCG) were purchased by Sigma-Aldrich (Sigma-Aldrich, St. Louis, MO).

**Cell lines.** A COS7 cell line permanently transfected with LHCGR (COS7/LHCGR) was kindly provided by Prof. Jöerg Gromoll (Centre for Reproductive Medicine and Andrology, University of Münster, Germany). The immortalized human granulosa cell line hGL5 [26] were permanently transfected by electroporation with LHCGR wild type. Each electroporations were performed by using 1x10^5^ cells resuspended in medium in the presence of 10 µg of plasmid, with the following settings: 430 Volt; 950 µF. Several hGL5 clones were selected for zeocin (Invitrogen, Leek, The Netherlands) resistance. The hGL5 clones were screened for *LHCGR* gene transcription by PCR, for LHCGR production by Western blot and for hLH and hCG responsivity in terms of cAMP production and ERK1/2 and AKT phosphorylation (data not shown), then only the better-responsive clones were cultured and used in our experiments. For stable transfection the pTracer vector (Invitrogen) was used for both cell lines, which contains the cytomegalovirus promoter in front of multiple cloning site and the green fluorescent protein reporter gene [18]. COS7/LHCGR cells were cultured in DMEM supplemented with 10% FBS, 2 mM L-glutamine, 60 µg/ml zeocin, 100 U/ml penicillin and 100 µg/ml streptomycin, at 37°C and with 5% CO_2_. This cell line, overexpressing the human LHCGR, was extensively validated previously [18]. hGL5/LHCGR cells were cultured in DMEM/F12 supplemented with 10% FBS, 2% Ultroser G, 2 mM L-glutamine, 80 µg/ml zeocin, 100 U/ml penicillin and 100 µg/ml streptomycin. All cell lines were maintained in incubator at 37°C and with 5% CO_2_.

**Granulosa-lutein cell isolation and culture.** Granulosa cells from 3-4 different patients were pooled and cultured in McCoy’s 5A medium supplemented with 10% FBS, 2 mM L-glutamine, 100 U/ml penicillin, 100 µg/ml streptomycin and 250 ng/ml Fungizone (all from Sigma-Aldrich), in well plates or culture slides (Nunc, Roskilde, Denmark). The number of cells and the feature of multi-well plates depends on the parameter to be evaluated.

**cAMP stimulation protocols.** The COS7/LHCGR, hGL5/LHCGR and granulosa cells were seeded in triplicate at a final concentration of 5x10^4^ viable cells/500 µl, in 24-well plates for experiments evaluating cAMP production. Cells were washed twice with PBS and serum starved 12 hours before the experiments. Then, a validated protocol was followed to perform the cAMP dose-response experiments [27]. Briefly, cells were stimulated using increasing doses of r-hLH, r-hCG, ex-hLH or ex-hCG as appropriate (0.1 pM-1 mM) diluted in 500 µl of medium without serum and pre-equilibrated at 37°C, then left under stimulation in the incubator in the presence of 500 µM phosphodiesterases inhibitor IBMX (Sigma-Aldrich). A negative control without gonadotropins and a positive control (50 µM Forskolin, Sigma-Aldrich) were also included. After 3 hours incubation, the entire well plates were frozen at -20°C until total cAMP measurement. Then, the cAMP ED_50_ values for hLH and hCG were calculated. A total of 4 independent experiments were performed.

To evaluate the kinetics of response to continuous exposure to gonadotropins, time-course experiments were performed. The COS7/LHCGR and hGLC were stimulated using the cAMP ED_50_ dose of recombinants hLH or hCG, previously calculated as above, diluted in 500 µl of medium without serum and left at 37°C and with 5% CO2 in the presence of IBMX, for different times ranging between 5 minutes and 36 hours. Negative and positive controls (without gonadotropins and in the presence of 50 µM Forskolin, respectively) were included for each time-step. After each incubation, the stimulating medium was quickly removed and well plates immediately frozen at -20°C, until intracellular cAMP measurement. A total of 3 independent experiments were performed. The cell viability was assessed by MTT assay (Promega, Madison, WI) during the 36 hours time-course experiments, as described below.

**cAMP Measurement.** The quantitative detection of cAMP was performed using the cAMP ELISA HTS Immunoassay Kit (Millipore, Billerica, MA) and evaluated by a multilabel plate reader (Victor3 from PerkinElmer, San Jose, CA), as indicated by the supplier. Total cAMP was measured in media containing extra- and intracellular cAMP, released from the cells after one cycle of freeze/thaw, while intracellular cAMP was measured only in cells treated with a lysis buffer included in the ELISA kit. Each sample was analyzed in triplicate against a cAMP standard dilution of 0-100 pmol/µl and evaluated by a luminometer capable of reading 96-well microplates (Victor3 from PerkinElmer). Lastly, the data were entered into a curve fitting software and represented using a log regression analysis.

**Cell viability assay.** Cell viability during time-course experiments was evaluated by MTT assay (data not shown). Human granulosa cells were cultured in 96-well plates, at density of 3x10^3^ cells/well. After culture for 6 days, the granulosa cells were treated with ED_50_ dose of hLH or hCG measured in terms of cAMP response, diluted in stimulating medium over 36 hours, while the cells without gonadotropin treatment served as control. The MTT assay was performed according to the procedure previously described [29] measuring the absorbance at wavelength of 560 nm using a microplate reader. A control without gonadotropins was also included at each time-step. Cell viability was expressed as the relative formazan formation in zearalenone-treated samples compared to control cells after correction for background absorbance.

**Immunoflorescence analysis of human granulosa cells.** Immunofluorescence analysis was performed to evaluate the kinetics of receptor internalization resulting from continuous *in vitro* stimulation of human granulosa cells by gonadotropins. Granulosa cells were seeded at 5x10^3^ cells/well in 4-wells slides and maintained at 37°C with 5% CO_2_. Six-days granulosa cells were serum-starved for 12 hours, then stimulated for different times (1, 8, 16, 24 hours) with the ED_50_ dose of hLH or hCG diluted in stimulating medium, and left in the incubator. A control without gonadotropins was also included at each time-step. After stimulation, the cells were immediately rinsed with PBS, fixed with ice-cold methanol, permeabilized and incubated with anti-LHCGR antibody (code #NBP1-04718; Novus Biologicals, Littleton, CO) overnight at 4°C (dilution 1:50 in PBS containing 0.1% BSA). The anti-LHCGR is an anti-peptide antibody previously tested for immunofluorescence and Western blot by a preabsorption with an excess peptide [30]. Cells were then incubated with secondary antibody TRITC-labeled anti-rabbit IgG (code #T6778; Santa Cruz Biotechnology, Santa Cruz, CA) at room temperature for 2 hours (dilution 1:200). Subsequently, the slides were rinsed with PBS and incubated 2 hours with anti-ERK1/2 antibody (dilutions 1:100), to allow the cytoplasmic co-localization of LHCGR, then incubated with secondary antibody FITC-labeled anti-rabbit IgG (code #F7512; Santa Cruz Biotechnology; dilution 1:200) and 4′,6-diamidino-2-phenylindole (DAPI) (Sigma-Aldrich) 50 ng/ml at room temperature for 2 hours. Western blot control for the anti-LHCGR antibody and non-permeabilized cells control were also included (Suppl. Fig. 5). Coverslips were mounted using 50% glycerol in PBS and observed using the confocal microscope DM IRE2 (Leica Microsystems, Wetzlar, Germany).

**Phospho-ERK1/2 and phospho-AKT stimulation and Western blot analysis.** The granulosa cells were seeded at a final concentration of 3x10^5^ viable cells/1 ml, in 12-well plates for the evaluation of ERK1/2- and AKT-pathways activation. Cells were washed twice with PBS and serum starved 12 hours before the experiments. To compare the response to recombinant *versus* extractive gonadotropins also hGL5/LHCGR cells seeded at the same conditions were used. To perform dose-response experiments evaluating the maximally stimulating doses (ED_MAX_), cells were stimulated for 15 minutes with increasing doses of r-hLH, r-hCG, ex-hLH or ex-hCG as appropriate (0.1 pM-1 mM) diluted in 1 ml of stimulating medium pre-equilibrated at 37°C and with 5% CO2, and left under stimulation in the incubator including negative controls without gonadotropins. 15 minutes is a common time to evaluate the ED_MAX_ for ERK1/2 and AKT stimulation by gonadotropins, as previously observed [31,32] and confirmed by our preliminary experiments (data not shown). Instead, in time-course experiments the cells were stimulated over 1 hours with the dose of hLH or hCG which determines the maximum level of stimulation of ERK1/2- and AKT-pathway, previously observed as above. The negative control consists in an unstimulated samples for each step of the time-course experiment. The reactions were stopped placing the entire well plates on ice and cells were immediately lysates for protein extraction in 4°C cold RIPA buffer added with phosphatase inhibitor cocktail PhosStop and protease inhibitor cocktail (Roche, Basel, Switzerland), 1.6 mM sodium orthovanadate and 1 mM phenylmethylsulfonyl fluoride (PMSF) (Sigma-Aldrich). A total of 4 independent experiments were performed. Each experiment were performed in a different pool of granulosa cells obtained from 3-4 different patients each time.

The protein content of cell lysates was determined and equal amounts of total proteins were subjected to 12% SDS-PAGE followed by Western blot analysis. The membranes were then incubated for 2 hours with antibody against phospho-ERK1/2 or phospho-AKT (codes #9101S and #9271S, respectively; Cell Signalling Technology, Boston, MA; 1:1000 dilution) at room temperature. Equal protein loading was confirmed in a stripped, washed and reprobed membrane with an antibody against total ERK1/2 (code #137F5; Cell Signalling Technology; 1:1000). The membranes were washed and incubated with horseradish peroxidase-conjugated secondary antibody (GE Healthcare, Little Chalfont, UK) for 1 hour at room temperature and signals were visualized using the ECL-Advance Western Blotting Detection Kit (GE Healthcare). Signals were acquired and semi-quantitatively evaluated by VersaDoc Imaging System and QuantityOne software (Bio-Rad Laboratories, Hercules, CA).

**Stimulation for gene expression analysis, total RNA extraction and cDNA synthesis.** The granulosa cells were seeded at a final concentration of 3x10^5^ viable cells/1 ml, in 12-well plates for gene expression analysis. Cells were washed twice with PBS and serum starved 12 hours before the experiments. The stimulations were performed incubating hGLC with r-hLH or r-hCG (100 pM) diluted in 1 ml of stimulating medium pre-equilibrated at 37°C and 5% CO2, and left under stimulation in the incubator. Where inhibitors (Sigma-Aldrich) were used, a one-hour pre-incubations of hGLC with 10 µM U0126 (ERK1/2-pathway inhibitor) or 20 µM LY294002 (AKT-pathway inhibitor) was performed. After one hour the solution was replaced with stimulating medium containing the gonadotropin. These inhibitors were used at known active concentrations [31]. Negative controls without gonadotropins were also included. After stimulation, total RNA was extracted from hGLC using Trizol reagent (Life-Technologies, Carlsbad, CA) following the manufacturers’ instructions. Quantification of total RNA was determined spectrophotometrically at 260 nm and RT used an equal amount of total RNA for each sample, avian myeloblastosis virus-reverse transcriptase and random hexamers (BioRad Laboratories, Hercules, CA) following the supplier’s instructions.

**Real-time PCR analysis.** Quantitative real-time RT-PCR was performed in a thermal cycler CFX96 (BioRad Laboratories) using SYBR green fluorescent detection system (Life-Technologies), according to the manufacturer’s recommendations. The expected PCR product length are shown in Table 1. Reactions were performed in triplicates using 5 μL 2X SYBR® Green PCR Master Mix (Applied Biosystems) in a final volume of 10 μL per reaction. All primers used in real-time PCR were designed using the Primer3 program (<http://frodo.wi.mit.edu/primer3>), verified by an online oligo analysis tool (www.operon.com by Eurofins MWG Operon, Huntsville, AL) and purchased from Integrated DNA Technologies (Coralville, IA). Prior to quantification by realtime RT-PCR, optimal primer concentration and annealing temperature were determined for each transcript, and the linearity of amplification for each target mRNA was similar to that of the endogenous control gene, ribosomal protein S7 (RPS7). The thermal cycling settings for all genes are the following: 45 cycles of 30 s of melting at 95°C followed by 10 s of annealing and extension at 60°C. After the amplification cycles, all samples were subjected to a melt curve analysis in which they were heated at 1°C/30 s increments from 61° to 94°C to validate the absence of non-specific products. Normalized gene expression was evaluated using the 2^-ΔCt^ method [33]. The final results obtained from each treatment were then expressed as fold increase over its unstimulated sample (basal level). A total of four experiments were performed.

**Statistical analysis.** Data are expressed as means ± SEM. To evaluate the statistical difference between hLH and hCG in cAMP dose-response and in gene expression experiments, the Mann Whitney’s *U*-test was performed. In time-course experiments, each data-set was verified with D’Agostino and Pearson normality test (alpha=0.05). For cAMP, each value obtained from hLH and hCG-stimulated cells was normalized for the corresponding control value measured at the same time-step and compared by unpaired T-test. In time-course experiments for ERK1/2 and AKT, the semi-quantitative evaluations were graphically expressed in relative units and each treatment was compared by Mann Whitney’s *U*-test *vs* control of the same time-point. Each value obtained from hLH/hCG-stimulated cells was then normalized to the respective control of the same time-point and the differences were evaluated by Mann Whitney’s *U*-test to compare results from several samples, and with two-way analysis of variance to compare entire data-set. Values were considered statistically significant for P<0.05. Statistical analysis were performed by GraphPad Prism software (GraphPad Software Inc., San Diego, CA).
